# Effectiveness of digital health applications on the quality of life in patients with overweight or obesity: a systematic review

**DOI:** 10.1186/s13690-024-01474-3

**Published:** 2025-01-09

**Authors:** Melanie Mäder, Tonio Schönfelder, Ria Heinrich, Carsta Militzer-Horstmann, Patrick Timpel

**Affiliations:** 1 Faculty of Economics and Management Science, Leipzig University, Chair for Health Economics and Management, Leipzig, Germany; 2 Scientific Institute for Health Economics and Health System Research (WIG2 GmbH), Markt 8, 04109 Leipzig, Germany; 3 Department of Health Sciences/Public Health, Dresden University, Dresden, Germany; 4 Fbeta GmbH, Berlin, Germany

**Keywords:** Digital health, Digital health applications, Digital health interventions, DiHA, DiGA, Overweight, Obesity, Quality of life, QoL

## Abstract

**Background:**

Globally, more than half of the adult population is overweight, including those who are obese, which increases the risk of premature death and reduces quality of life (QoL). Technologies such as digital health applications (DiHA) can potentially improve clinical outcomes (e.g., health status, illness duration, QoL) or patient-related factors (e.g., therapy monitoring, adherence, health literacy). To date, there is no systematic review addressing the effectiveness of DiHA on the QoL in patients with overweight or obesity.

**Objective:**

The objective was to investigate the impact of DiHA on QoL in overweight or obese patients.

**Methods:**

A systematic literature search was conducted in MEDLINE via PubMed, Cochrane Library, and Embase via Ovid in 2023, supplemented by additional manual searches. The eligibility criteria included patients with overweight and/or obesity who used a digital intervention independently and without interaction with a healthcare professional. The outcome of interest was QoL. As potentially eligible trials had to demonstrate effectiveness, only randomized controlled trials (RCT) were included as the minimum evidence standard. The study screening (title-abstract, full-text) was conducted independently by two researchers using pre-specified eligibility criteria. CONSORT-EHEALTH checklist was used for data extraction of qualitative and quantitative data (study characteristics and study results) and the Cochrane Risk of Bias Tool (version 2) for quality assessment independently by two researchers.

**Results:**

Seven RCT conducted in Europe and the United States were included in this systematic review with a total sample size of *N* = 946. Observation periods were heterogeneous and ranged from 3 to 24 months. The evaluated interventions consisted of websites or apps, all of which included nutrition and physical activity features, and functioned independently with minimal or no involvement of a healthcare professional. All studies showed a high risk of bias, no statistically significant improvement and no effects regarding QoL using different validated questionnaires.

**Conclusions:**

This systematic review provides a comprehensive analysis of DiHA effectiveness on QoL in patients with overweight or obesity. Overall, there is heterogeneity regarding the operationalization of QoL and the examined interventions have no statistically significant impact on QoL. Comparable systematic reviews show that digital interventions have the potential to improve the QoL of these patients, but further RCT and high-quality studies are needed to assess the impact of DiHA on QoL.

**Trial registration:**

PROSPERO CRD42023408994.

**Supplementary Information:**

The online version contains supplementary material available at 10.1186/s13690-024-01474-3.

**Table:** 

Text box 1. Contributions to the literature
• Evidence on the impact of digital health applications (DiHA) on quality of life (QoL) in patients with overweight/obesity is limited.
• Based on the identified seven eligible studies, available DiHA evidence does not support a benefit in terms of improved QoL in patients with overweight/obesity.
• However, it is likely that short observation periods, insufficient sample sizes and the use of generic rather than disease-specific QoL instruments may have contributed to the inconclusive evidence.
• Our findings help to develop DiHA with focus on improving patient’s QoL and to decide on characteristics for the design of future DiHA studies.

## Introduction

In 2022, around 16% of adults worldwide suffered from obesity and the global prevalence more than doubled between 1990 and 2022 [1]. Obesity constitutes a risk factor for secondary diseases such as type 2 diabetes mellitus, cardiovascular diseases, and certain cancers. The risk of other diseases increases with elevated severity [2]. Every year, more than 5 million people die due to comorbidities originally caused by overweight and obesity [3]. Of total healthcare expenditure in Organization for Economic Cooperation and Development countries, 8.4% is allocated to the treatment of obesity-related diseases [4]. In Europe, direct costs due to overweight and obesity of US$ 195.53 billion are expected by 2030 and direct costs of US$ 347.89 billion by 2060 [5]. Additionally, obesity increases the risk of premature death and diminishes the quality of life (QoL) [6].

Digital health interventions have the potential to enhance QoL [7], improve therapy monitoring, or increase therapy adherence [8], and are available in different forms. Digital health interventions (e.g., electronic medical records, mobile applications, wearable sensors for remote monitoring) are healthcare services that are delivered electronically as part of formal or informal care [9]. Digital health applications (DiHA), approved by the German Federal Institute for Pharmaceuticals and Medical Products, represent one type of these digital health interventions. DiHA have already been implemented in standard care in other countries such as Belgium, France and Austria [10]. As certified medical devices of low or middle-risk class, DiHA have been part of statutory standard care in Germany since 2020. German DiHA can be mobile applications or web applications whose primary function is digital, and whose medical purpose is achieved through the main digital function. They are used to detect, monitor, treat, or alleviate illnesses, injuries, or disabilities, primarily by patients. DiHA are primarily used by the patients directly by interacting with them, while allowing the healthcare professionals to communicate with the patients. Applications that are used exclusively by healthcare professionals for the treatment of patients (e.g. telemedicine applications for video consultations) or that only read other devices such as smartphones and transmit data (e.g. smartwatch, digital personal scales) are not DiHA. A key criterion for inclusion in statutory standard care is proof of a positive healthcare effect. A special feature of DiHA is that the patient-relevant, positive healthcare effect is achieved by the DiHA itself and not by the influence of a healthcare professional or an accompanying therapy.

One option is the improvement of QoL by the DiHA [11]. To date, 56 DiHA are listed in the German DiHA directory, of which 38 address QoL [12]. Two of these applications (Oviva Direkt für Adipositas and zanadio) were developed for patients with overweight/obesity [13, 14]. QoL plays a significant role in medical decision-making, serves as a strong predictor of survival after an illness, and can influence the success of treatment [15]. The WHO defines QoL as "an individual's perception of their position in life in the context of the culture and value systems in which they live and in relation to their goals, expectations, standards and concerns" (WHO 1995). In recent decades, research has increasingly focused on patients’ QoL, and its assessment has become increasingly important [16]. Nevertheless, there is no standardized definition of QoL, which leads to different ways of operationalizing QoL [15]. For this reason, researchers have developed core outcome sets (COS)—agreed-upon, standardized outcome groups that enable consistent and transparent reporting of results from studies in a defined clinical area [17]. The development of COS has taken place in the context of behavioral weight management programs for adults with overweight and obesity, which also encompass QoL defined as a measure of participants' overall well-being [18].

As part of a systematic review (*n* = 303), Knapp et al. examined the utilization of patient-reported outcome measures and patient-reported experience measures in evaluation studies of telemedicine applications. QoL was the most frequently addressed outcome across all studies [19]. Dehghan Ghahfarokhi et al. examined the effectiveness of wearables and smartphone applications on physical activity, QoL, and cardiovascular outcomes in overweight/obese adults as part of a systematic review (*n* = 26). The authors' results showed a significant improvement in QoL (standard mean difference (SMD) = 0.33; 95% confidence interval (CI) = 0.14–0.52; *p* < 0.001) [20]. However, the available evidence syntheses either focused on telemedicine or used an activity tracker to increase physical activity, neither of which are in line with the definition of DiHA. Haraldstad et al. conducted a systematic review (*n* = 163) of research on QoL in medicine and health sciences. The authors' findings show that QoL research addresses various target groups, utilizes diverse research designs, and employs different operationalization tools. The authors emphasize the need for further research into the design of studies on QoL, which should give greater consideration to methodological and conceptual aspects [16].

These studies [16, 19, 20] call for further research, especially providing relevant design characteristics for studies that have been established and can be used for the design of future comparative DiHA studies which focus on QoL. The focus of previous systematic reviews on the effectiveness of digital interventions in patients with overweight/obesity is on medical endpoints such as BMI and weight and thus less on what weight loss means for the patient themselves. This is why patient-reported outcomes (PROs), such as QoL, are becoming increasingly important within healthcare research [19]. Although studies have shown that weight loss improves obesity-associated comorbidities, there is hardly any evidence about the effect of weight loss on QoL [21]. In the absence of a systematic review studying the specific impact of DiHA on QoL in patients with overweight or obesity, and limited knowledge on how to design high-quality trials studying the effectiveness of DiHA, the present systematic review aims to close this gap in research. This systematic review also raises awareness of patient-reported outcomes and their importance.

### Research questions

The following research questions are addressed in the systematic review:

Primary research question:

What is the impact of DiHA on QoL in patients with overweight or obesity?

Explorative research question:

Which study-relevant design characteristics have been established and can be used for the design of future comparative DiHA studies?

## Methods

Prior to commencing the systematic review, a search for comparable reviews was conducted via the International Prospective Register of Systematic Reviews (PROSPERO) on 11/02/2023. No review addressing the above research questions could be identified. This systematic review was registered in PROSPERO on 03/17/2023 under the number CRD42023408994.

### Search strategy

The systematic literature search was performed in the databases MEDLINE via PubMed, Cochrane Library, and Embase via Ovid, following the established guidelines for conducting and reporting systematic reviews (Preferred Reporting Items for Systematic Reviews and Meta-Analyses; PRISMA) [22]. Initially, various search terms for the individual PICO(S) elements were identified in the form of keywords (MeSH terms, Emtree terms: adiposity, obesity, overnutrition, overweight, cell phone, digital technology, electronic mail, mobile applications, text messaging, virtual reality, quality of life), text words/keywords, and linked using the Boolean operator "OR". Subsequently, the individual PICO(S) elements regarding "population", "intervention" and "outcome" were connected using the operator "AND". An individual search strategy was developed, which was adapted to the database-specific requirements of the respective database (see Additional file 1). The devised individual search strategy used: (1) relevant systematic reviews in this area [19, 20, 23, 24], (2) recommendations from the Cochrane Handbook for Systematic Reviews of Interventions [25] and (3) recommendations from the InterTASC Information Specialists' Sub-Group [26]. In addition to the database searches, extensive manual searches were conducted to complement the research process:

- Search for "grey literature" in non-formally published sources (e.g., Google Scholar, conference proceedings, scientific articles in pre-publication, research papers) [25].
- Forward search: search for further relevant studies in the reference lists of the included studies [27].
- Backward search: search for further relevant studies that have already cited the included studies in Google Scholar [27].
- Search for further relevant studies in these systematic reviews and meta-analyses which were identified through the search string.

### Eligibility criteria

Leading up to the search, specific inclusion and exclusion criteria were defined following the PICO(S) scheme (Table 1). The criteria were used for both title-abstract screening and full-text screening to select appropriate studies. The defined study population focused on individuals with overweight (25 to less than 30 kg/m^2^) or obesity (≥ 30 kg/m^2^) [28]. The studies were included if the average BMI and the BMI range were within this defined range. Digital interventions were eligible for inclusion if their primary function was based on digital technologies. In contrast, analog interventions, or digital interventions whose effect was not primarily due to digital technology but, for example, demonstrated a significant impact of a healthcare professional, were excluded. Only this specific definition of the intervention makes it possible to answer the research questions and to ensure that possible effects in relation to QoL can be assessed. Studies were included if they assessed QoL as a primary or secondary outcome. Consequently, studies in which QoL was not, or only exploratory assessed, were excluded. Studies in parallel group design, e.g., randomized controlled trials, controlled trials, or studies in cross-over design were eligible for inclusion. In contrast, systematic reviews, or meta-analyses as well as observational studies, expert opinions or discussion papers, study protocols, and pilot/feasibility studies were excluded. Identified evidence syntheses were assessed for included primary studies (manual search).

**Table 1:** Inclusion and exclusion criteria for database search

PICO(S)-Criteria	Inclusion criteria	Exclusion criteria
Patient	Humans	Animals
	Overweight: BMI ≥ 25.0 – 29.9 kg/m^2^	Pregnancy and/or breastfeeding
	Obesity: BMI ≥ 30 kg/m^2^: ▪ Severity 1: BMI 30.0 – 34.9 kg/m^2^ ▪ Severity 2: BMI 35.0 – 39.9 kg/m^2^ ▪ Severity 3: BMI ≥ 40.0 kg/m^2^	Certain comorbidities (e.g., depression, diabetes), unless the primary reason for treatment is overweight or obesity (i.e., digital interventions examined in the studies should specifically address overweight/obesity)
Intervention	Digital health application or comparable intervention whose function meets the requirements of a DiHA:	Digital intervention whose function does not meet the requirements of a DiHA:
	-main function is based on digital technologies	-main function is not based on digital technologies
	-telemedicine applications as a component of an application, if the main function is predominantly based on digital technologies	-exclusively telemedical platform
	-assists in the detection, monitoring, treatment, or mitigation of disease, injuries or disabilities	-applications that serve primary prevention
	-use by the patient or shared use by health care professionals and patients	-applications used solely by the physician for the treatment of patients (practice equipment) -applications whose positive healthcare effects are not primarily attributable to the digital technology (but, for example, to the healthcare professional)
	-patient interaction with the application	-applications that only read other devices such as smartphones and transmit data
Control	Usual care	No control group
	No treatment	
	Waiting list	
	Treatment with a comparable DiHA	
Outcome	Quality of life (primary or secondary endpoint)	Quality of life is either not surveyed or is surveyed only exploratively
Study Design	Randomized controlled trials	Systematic reviews, meta-analyses
	Controlled trials	Observational studies (case series or reports, cross-sectional studies, case–control studies, cohort studies)
	Cross-over studies	Expert opinions, discussion papers
		Study protocols
		Pilot and feasibility studies, which were explicitly designated as such
Language	German or English	All other

*BMI* Body mass index, *DiHA* Digital health application

### Title-abstract- and full-text screening

After removing duplicates (software used: Citavi 6.18.0.1), the identified records underwent screening at the title-abstract level (software used: Rayyan), by applying the eligibility criteria. The resulting relevant references were subsequently screened at full-text level. These screening processes were independently conducted by two reviewers (MM and PT). Studies and references with any disagreement or uncertainty underwent further review in the next step and were discussed collaboratively. If no consensus could be reached, a third, independent person was consulted, and a collective decision was made.

### Data extraction

For data extraction, a pre-tested structured extraction sheet following the CONSORT-EHEALTH checklist (V.1.6.1) was used to extract the following qualitative and quantitative data: (1) author, (2) title, (3) inclusion and exclusion criteria, (4) gender, (5) age, (6) BMI, (7) intervention, (8) intervention support, (9) control, (10) endpoints and operationalization, (11) study design, (12) randomization sequence, (13) stratification, (14) blinding, (15) observation period, (16) sample size, (17) drop-out, (18) place of the study, (19) time of the study. Additionally, detailed extraction of study results on QoL and all primary endpoints was performed.

### Quality assessment

The study quality of RCT was assessed using the Cochrane Risk of Bias Tool version 2 (RoB2) [29]. The RoB2 tool identifies possible sources of bias that may arise in the following areas: (1) randomization process (selection bias), (2) deviations from intended interventions (performance bias), (3) missing outcome data (attrition bias), (4) measurement of the outcome (detection bias) and (5) selection of the reported results (reporting bias). The ratings from these five domains were utilized to determine an overall risk of bias rating for the study, in relation to the outcome of interest (QoL). The response options for the overall judgment align with those for the individual domains, namely "low risk of bias," "some concerns," "high risk of bias". If the study's risk of bias concerning QoL was assessed as high in at least one domain or with some concerns in multiple domains, then a high risk of bias was assigned [30]. Quality assessment for the impact of assignment to the intervention was conducted in parallel and independently by two researchers (MM and PT). Any disagreements or uncertainties led to a reevaluation and discussion of the studies. If no consensus could be reached, a third, independent party was consulted, and a collective decision was made.

### Data synthesis and analysis

The presentation of the results was descriptive. Effect sizes were calculated based on the respective sample size, mean values, and standard deviations. A prerequisite for conducting a meta-analysis is the existence of at least five comparable studies [31]. The comparability of these studies was independently assessed in parallel by two researchers based on (1) population characteristics (age, gender), (2) intervention details (intervention character, intervention duration), and (3) operationalization of the outcome QoL (questionnaire used, observation duration). Effect sizes according to Cohen's d were calculated for studies in which such measures were provided. For this purpose, the difference between the mean values of the intervention group (IG) and the control group (CG) was divided by the standard deviation in studies with the same group sizes, assuming a t-test was conducted. Interpretation of effect sizes according to Cohen's d was as follows: 0 to < 0.2 indicates no or very small effect; 0.2 to < 0.5 signifies a small effect; 0.5 to < 0.8 suggests a medium effect and ≥ 0.8 represents a strong effect [32].

## Results

### Review characteristics

**Fig. 1:**
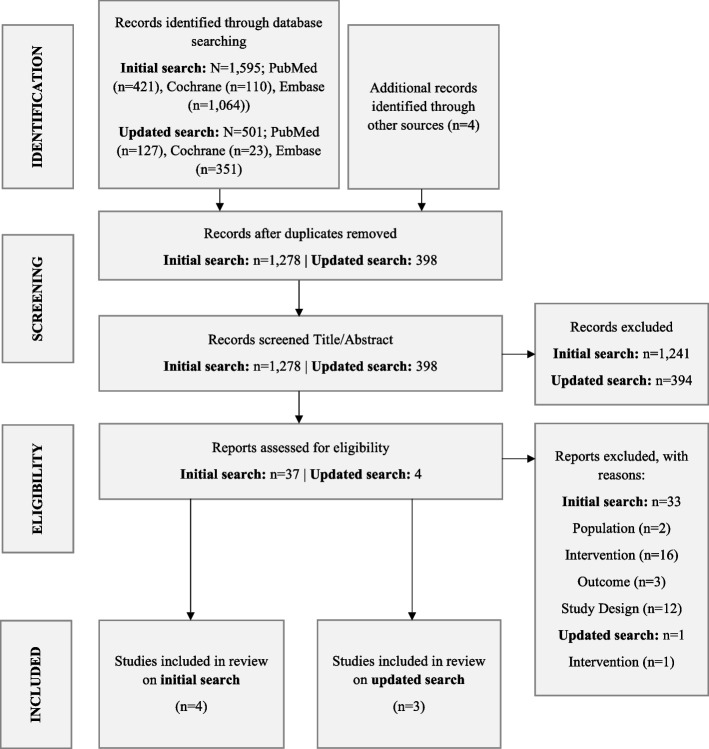
PRISMA (Preferred Reporting Items for Systematic Reviews and Meta-Analyses) flow chart depicting study inclusion

Initially Database searches (performed on 21/03/2023) yielded a total of *n* = 1,595 results. Additionally, *n* = 4 studies were identified through other sources. Following the removal of duplicates, *n* = 1,278 results were available for title-abstract screening. Out of these, *n* = 1,241 studies were excluded during title-abstract screening phase. The remaining *n* = 37 studies underwent full-text screening and were assessed for eligibility. After excluding non-eligible records during full-text assessment (*n* = 33), four studies remained for further analyses. The primary reasons for exclusion during full-text screening were interventions (*n* = 16), study design (*n* = 12), outcomes (*n* = 3) or population (*n* = 2) not aligning with the pre-defined inclusion criteria. Most studies were excluded due to the requirement of direct involvement of a healthcare professional in the studied intervention, which did not meet the specified criteria for a DiHA. Studies excluded based on study design were conference abstracts, study protocols, or pilot studies. Results excluded due to outcome did not examine QoL, and studies excluded because of population also included patients with BMI < 25 without evaluating them in subgroup analysis. A comprehensive list of excluded studies with reasons for exclusion can be found in Additional file 2. The search was updated in September 2024 and *n* = 501 studies could be identified through the three databases (PubMed = 127, Cochrane Library = 23, Embase = 351), of which three met the eligibility criteria and were included in the systematic review. Finally, a total of seven studies were included in the qualitative data synthesis. Figure 1 illustrates the process of study selection.

### Study characteristics

The included studies were conducted in Spain (*n* = 2), USA (*n* = 2), Germany (*n* = 2), and the UK (*n* = 1). All studies were published between 2007 [33] and 2023 [13, 14]. There were conflicts of interest for the two studies on the obesity applications already listed in the German DiHA directory [13, 14], as the study authors were also the founders or employees of the respective application. All the included studies were RCT with a 1:1 randomization into an IG and a CG. The observation period ranged from 3 months (*n* = 4) to 24 months (*n* = 1); in the remaining studies (*n* = 2), a period of 12 months was chosen. The frequency of QoL measurement was heterogeneous and occurred at two [34, 35], three [13, 33, 36], four [37] or five [14] time points. Sample size calculations were carried out in all studies and sample sizes ranged from 56 patients [36] to 221 patients [33], with an overall sample size of all included studies of *N* = 946. Drop-out varied from 0% [36] to 50% [35], with an overall drop-out rate for all included studies of 16.98%. Drop-out reasons were not given in three studies [34, 36, 37]. In the remaining studies, the reasons given for drop-outs were: lack of contact possibility, relocation, lack of participation in the visit, withdrawal of participation in the study, discontinuation of the intervention, pregnancy or health reasons [13, 14, 33, 35]. The lowest mean age reported was 43.4 (10.9) years [14], while the highest mean age reached 57.0 (10.1) years [35]. Overall, the studies included 593 female participants and 241 male participants. In the study of Mensorio et al., no information was provided regarding the gender of the participants. The lowest mean BMI observed was 29.6 (3.5) [35], categorized as overweight, while the highest mean BMI recorded was 36.1 (7.6) [36], corresponding to obesity severity level two. Adherence or usage data were collected in four of the included studies [13, 33–35]. All studies imposed age restrictions, with three studies considering the age group of 18–65-year-olds [14, 33, 37]. An overview of the complete study and population characteristics of the included studies is presented in Table 2. A complete list of the study characteristics can be found in Additional file 3.

**Table 2:** Study and population characteristics of the included studies

Author (Year)	Mensorio et al. (2019) [37]	Mangieri et al. (2019) [36]	Kraschnewski et al. (2011) [34]	McConnon et al. (2007) [33]	Roth et al. (2023) [14]	Múzquiz-Barberá, et al. (2023) [35]	Gemesi et al. (2013) [13]
Country	Spain	USA	USA	UK	Germany	Spain	Germany
Study design (randomization sequence)	RCT (1:1)	RCT (1:1)	RCT (1:1)	RCT (1:1)	RCT (1:1)	RCT (1:1)	RCT (1:1)
Observation times in months	3^A^; 6; 12	12; 24^A^	3^A^	6; 12^A^	3, 6, 9, 12^A^	3^A^	3^A^, 6
Sample size (n)	106	56	100	221	150	132	181
Drop-Out ^A^ (n (%))	15 (14.15%)	0 (0.00%)	12 (12.00%)	21 (9.50%)	15 (10.00%)	66 (50.00%)	42 (23.20%)
Age (M (SD)) in years	53.00 (8.90)	52.75 (9.80)	50.30 (11.00)	45.80 (10.60)	43.40 (10.90)	56.95 (10.10)	46.880 (11.00)
Gender (n)	n.r.	f = 50 m = 6	f = 69 m = 31	f = 170 m = 51	f = 136 m = 13	f = 60 m = 72	f = 108 m = 60
BMI (M (SD)); kg/m2	30.12 (2.80)	36.12 (7.59)	33.20 (4.20)	34.45 [31.85–38.70]^B^	35.80 (3.20)	29.6 (3.45)	34.20 (2.80)

^A^Primary survey time point

^B^Specified as median [interquartile range]

*BMI* body mass index | f = female | *m* = male | *M* = mean | *n* = absolute frequency | *n.r.* = not reported | *RCT* randomized controlled trial | *SD* standard deviation

### Intervention characteristics

The included studies investigated the effectiveness of digital interventions, comprising either websites (*n* = 4) or apps (*n* = 3). Notably, the intervention Living Better was explored in two studies, each with distinct objectives. The intervention includes digital behavioral therapy in relation to eating habits and physical activity with the aim of weight reduction. Mensorio et al. scrutinized the efficacy of the Living Better intervention, while Múzquiz-Barberá et al. slightly modified it in their efficacy study. In the IG, the participants’ hypertension specialist presented audiovisual material of the website, whereas in the CG, the presenting physician was not known to the participants. Two interventions, zanadio and Oviva Direkt für Adipositas, are already permanently listed as DiHA in the German DiHA directory [13, 14]. Additionally, one intervention, MyFitnessPal, was used for patients post-bariatric surgery [36].

All interventions addressed the topics of nutrition and physical activity. Two interventions, Living Better and zanadio, [14, 35, 37] also contained behavioral therapy approaches.

In two studies, the digital intervention served as a supplement to standard care with minimal (in five studies) or nonexistent (in two studies) support from a healthcare professional. When present, minimal support involved technical assistance or introduction to the digital intervention. In the study by Mensorio et al., participants were contacted only if no modules were accessed for more than two weeks. CG in four of the included studies received standard care, while in two studies, a wait-list control group was designed, allowing access to the study after the observation period concluded. In one study, the CG also received a digital intervention; however, unlike the IG, an unknown physician presented the audiovisual material. The duration of the interventions varied, ranging from 3 months (*n* = 4) to 12 months (*n* = 2), to 24 months (*n* = 1) (see Table 3).

**Table 3:** Intervention characteristics of the included studies

Author (Year)	Mensorio et al. (2019) [37]	Mangieri et al. (2019) [36]	Kraschnewski et al. (2011) [34]	McConnon et al. (2007) [33]	Roth et al. (2023) [14]	Múzquiz-Barberá, et al. (2023) [35]	Gemesi et al. (2023) [13]
Intervention	Website	App	Website	Website	App	Website	App
Intervention group	Living Better: -9modules -behavioral therapy, eating habits, physical activity -psychoeducation about healthy lifestyle, learning techniques -self-observation, self-instruction, behavioral recording, stimulus control, self-reinforcement, problem-solving + UC	MyFitnessPal: -nutrition diary -comparison of the number of calories with previously set goals -training diary for documenting training duration, intensity and number of calories burned -progress overview + standard post-operative care	AchieveTogether: -36 weight-control behaviors -target body weight -documentation of weight, height, and frequency of using the 36 weight-loss practices in the past 7 days -weight-loss plans -weekly goals	-dietary advice, physical activity advice, behavior therapy -enablement to manage own care -personalized advice -specific motivational statements -details of progress (self-reported weight loss)	zanadio: -multimodal approach including validated methods from behavioral science, exercise therapy, and nutrition -different features: knowledge transfer, change, motivation, and support	Living Better with own hypertension specialist	Oviva Direkt für Adipositas: -multimodal weight loss intervention program -main elements: self-management, self-monitoring, and education -daily/weekly goals -data documentation -automated feedback
Support	-reminder E-Mail if participants stop accessing modules for > 2 weeks	-reminders for app use -technical support	-demonstration of the website and its services	-demonstration of the website and its services	none	none	-demonstration of the app and its services
Control group	UC: -information on lifestyles and diet -Living Better after 3 months	UC: -self-monitoring journals + standard post-operative care	wait-list; website access after 12 weeks	UC: -printed information	UC: -self initiated or externally initiated weight loss attempts	Living Better with unknown physician	wait-list; app access after 12 weeks
Intervention duration in months	3	24	3	12	12	3	3

*UC* Usual care

No adverse events were reported in any of the included studies in relation to the intervention investigated.

### Quality assessment

The quality assessment conducted with the RoB 2 tool revealed a high risk of bias for all the included studies. Specifically, four studies [13, 33, 34, 37] demonstrated a high risk of bias in more than one domain. In the study by Mangieri et al., a high risk of bias was observed in only one domain, but three other domains were assessed with some concerns [36]. The overall high risk of bias is mainly due to a high risk of bias within domains two "deviations from intended interventions" and three "missing outcome data". Risk of bias within domain two was appraised with some concerns in one study [37] and as high in the remaining six studies. The primary sources of bias within this domain included (1) participants being aware of their assigned intervention during the trial in all the included studies, (2) carers and individuals delivering the interventions were aware of participants' assigned intervention during the trial, or the absence of information about this fact, and (3) the lack of appropriate analysis (e.g., intention-to-treat analysis, ITT) used to estimate the effect of assignment to intervention. Risk of bias within domain three was assessed as low in three studies [14, 35, 36] and high in four studies [13, 33, 34, 37]. The primary sources of bias within this domain included (1) unavailability of data for the QoL outcome for all or nearly all, participants randomized and (2) lack of evidence ensuring that the result was not biased by missing outcome data (e.g., ITT analysis). An overview of the core results of the quality assessment can be found in Table 4. The detailed quality assessment of the individual studies is available in Additional file 4.

**Table 4:** Quality assessment of the included studies

Author (Year) RoB2 Domain	Mensorio et al. (2019) [37]	Mangieri et al. (2019) [36]	Kraschnewski et al. (2011) [34]	McConnon et al. (2007) [33]	Roth et al. (2023) [14]	Múzquiz-Barberá, et al. (2023) [35]	Gemesi et al. (2023) [13]
Randomization process							
Deviations from intended interventions							
Missing outcome data							
Measurement of the outcome							
Selection of the reported result							
Overall risk of bias							

If the study's risk of bias related to quality of life was assessed as high in at least one domain or with some concerns in multiple domains, then there is a high risk of bias [22]

### Study results-Quality of life

All studies utilized validated questionnaires to assess QoL, employing varied instruments such as the Quality of Life Index; Spanish version [37], Research and Development-36 [36], Impact of Weight on Quality of Life questionnaire [34], European Quality of Life questionnaire [13, 33], World Health Organization Quality of Life-BREF [14] and SF-12 Health Questionnaire [35]. Notably, no statistically significant difference emerged between IG and CG in any of the included studies. Furthermore, all effect sizes fell within the range of 0 to < 0.2, indicating no discernible effect. It is important to note that a specific effect size could not be computed for the study by Mangieri et al. [36] due to insufficient information on mean values and standard deviations (see Table 5). None of the included studies used clinical relevance margins to assess the clinical relevance of the QoL-results obtained for patients. Given the heterogeneity among the included studies, particularly in terms of the operationalization of QoL and the duration of observation, conducting a meta-analysis was not feasible.

**Table 5:** Study results of the included studies – QoL

**Author (Year) **Methods of analysis	**Statistical measure**	**Operationalization**	**Observation time**	**Results IG**	**Results CG**	*p* **-value**	**Effect size**	**Risk of Bias**
Mensorio et al. (2019) CG received the digital intervention after 3 months ANOVA, ITT [37]	M (SD)	Quality of Life Index, Spanish version [60]	t0 (baseline)	7.01 (1.34)	7.17 (1.12)	.08		High
			t1 (3 months)^A^	7.15 (1.24)	7.13 (1.3)	.36	0.016^B^	
			t2 (6 months)	6.93 (1.1)	7.76 (1.0)	n.r.		
			t3 (12 months)	6.96 (1.0)	n.a.	n.a.		
Mangieri et al. (2019) ANOVA [36]	M (SD)	Research and Development-36 [61]	t0 (baseline)	n.r.	n.r.	not significant	n.p.	High
			t1 (12 months)	n.r.	n.r.			
			t2 (24 months)^A^	n.r.	n.r.			
Kraschnewski et al. (2011) ANCOVA [34]	M [95% CI]	Impact of Weight on Quality of Life questionnaire [62]	t0 (baseline)	n.r.	n.r.	n.r.		High
			t1 (3 months)^A^	2.6 [−0.2, 5.4]	0.6 [−2.2, 3.5]	not significant	0.195^B^	
Mc Connon et al. (2007) ANCOVA [33]	Median [IQR]	European Quality of Life questionnaire [63]	t0 (baseline)	70 [55 to 80]	65 [50 to 80]	n.r.		High
			t1 (12 months)^A^	n.r.	n.r.	.8	0.062^B^	
Roth et al (2023) ITT [14]	M (SD)	World Health Organization Quality of Life-BREF [64]	t0 (baseline)	55.6 (18.0)	54.6 (16.7)	n.r.		High
			t1 (12 months)^A^	MD = 1.60 [−3.55 to 6.75]		.543	0.06^B^	
Múzquiz-Barberá et al. (2023) ANOVA, ITT [35]	M (SD)	SF-12 Health Questionnaire [65]	t0 (baseline)	40.1 (22.8)	46.9 (15.1)	.061		High
			t1 (3 months)^A^	45.3 (21.6)	48.8 (15.8)	.328	0.029^C^	
Gemesi et al. (2023) Completers-Analysis [13]	M (SD)	European Quality of Life questionnaire, EQ-5D-5L Index [63]	t0 (baseline)	0.94 (0.1)	0.96 (0.1)	.33		High
			t1 (3 months)^A^	− 0.02 (0.1)	− 0.001 (0.1)	.32	0.190^B^	

^A^Primary survey time point

^B^Effect size Cohens d

^C^Effect size Partial eta^2^

*ANCOVA* analysis of covariance | *ANOVA* analysis of variance | *BMI* body-mass-index | *CG* control group | *CI* confidence interval | *IG* intervention group | *IQR* interquartile range | *ITT* intention-to-treat | *M* mean | *MD* mean difference | *n.a.* not applicable | *n.p.* not possible | *n.r.* not reported | *SD* standard deviation | *QoL* quality of life

In the Quality of Life Index, Spanish version, the total score ranges from 1–10; in all other questionnaires, the total score ranges from 0–100. In all the questionnaires used, higher values are associated with better QoL

### Study results-Behavior change

Four of the included studies investigated a possible behavioral change of the study participants in relation to weight-control behaviors [34], dietary habits and eating behaviors [33, 37] or physical activity [33, 35, 37]. Only two studies [34, 37] showed a statistically significant difference between IG and CG at the primary survey time point according to behavior change endpoints.

### Study results – BMI and weight

The primary outcome in four of the included studies focused on weight loss [13, 14, 33, 34]. Conversely, the remaining studies investigated BMI [35, 37] and the percentage of excess bodyweight loss, along with the percentage of excess BMI loss [36] as their primary endpoints. Five studies [13, 14, 34, 36, 37] demonstrated that the digital intervention led to a statistically significant improvement in IG compared to the CG, concerning the primary endpoint at the primary survey time point. The complete study results on BMI and weight can be found in Additional file 5.

### Main findings

All studies conducted a non-blinded RCT comparing an IG with a CG. Observation times ranged between three to 24 months. Sample sizes ranged from 56 to 221 patients, based on statistical sample size calculation. The overall drop-out rate was 17%. All studies examined QoL as secondary endpoint by using different validated and mainly generic questionnaires. Since all included studies did not show statistically significant changes in QoL, it is not possible to derive study-relevant design characteristics for the design of future comparative DiHA studies based on the results of the systematic review alone.

## Discussion

The scientific literature on obesity highlights that there is very limited evidence for mental health outcomes such as QoL [38]. For this reason, a systematic literature review examining the best available evidence to discern the impact of DiHA on the QoL in patients with overweight or obesity was conducted. All included studies assessed QoL as a secondary endpoint, employed different validated questionnaires, and none of them found statistically significant differences in QoL between IG and CG. Although all included studies were RCT, risk of bias assessment showed high risk of bias for all the included studies.

### Principal results

None of the included studies found statistically significant improvements in QoL in patients with overweight or obesity following DiHA use. The correlation between weight loss and QoL remains inconclusive in the scientific literature. Some studies suggest that the changes in QoL were independent of weight loss [39, 40] while others indicate that changes in QoL were a consequence of weight loss [41, 42]. Stephenson et al. identified an association between increasing weight status and decreasing QoL using a long-term cohort study, especially in people within the highest BMI category [43]. Warkentin et al. also investigated the effect of weight loss on QoL as part of a meta-analysis and found that there was no statistically significant association between weight loss and an overall improvement in QoL. Small effects were identified regarding improvements in physical health, but not in mental health [44].

A systematic review of reviews conducted by Kolotkin and Andersen [45] assessed the relationship between obesity, weight loss and health-related quality of life. The authors assert that establishing a clear, proven association between weight loss and enhanced QoL based on existing RCT is challenging. This difficulty arises from the diverse survey instruments employed, the heterogeneity in the studied interventions and their individual functions, inadequate statistical power within the studied groups, and the insufficient recording of QoL results. There are some possible reasons potentially explaining the lack of statistically significant differences within QoL in this systematic review, which should be specifically considered in future studies in this subject area:

1. All the included studies in this systematic review adopted a RCT design with a 1:1 randomization sequence into an IG and a CG. Due to the nature of digital interventions, blinding of participants was not feasible in any of the included studies. Notably, blinding in digital intervention studies is only achievable by incorporating a placebo application [46]. Nevertheless, blinding of the persons processing the data was ensured.
2. Observation periods across the studies were diverse, ranging from three to 24 months. However, it is important to acknowledge that excessively short follow-up periods can hinder the derivation of meaningful conclusions about long-term outcomes [47].
3. Sample sizes were heterogeneous, varying from 56 to 221 subjects. While the calculation of the optimal sample size ensures sufficient power for determining statistically significance [48], it is noteworthy that some studies may not have been adequately powered to identify statistically significant differences in QoL, although sample size calculation was carried out in all the included studies.
4. It is noteworthy that in most studies weight loss, not QoL, serves as the primary endpoint [45]. This observation aligns with our findings, where all included studies investigated QoL as a secondary endpoint.
5. Despite the consistent use of validated questionnaires across the included studies to operationalize QoL, different questionnaires were employed. Numerous survey instruments categorize QoL into generic, obesity-specific, or combined generic/obesity-specific forms. One widely used generic instrument [45] in the context of overweight and obesity is the Short-Form-36 (SF-36), utilized in its original version by Mangieri et al. The most frequently used obesity-specific instrument [45] is the Impact of Weight on Quality of Life-Lite (IWQOL-Lite), also employed by Kraschnewski et al. Kolotkin and Andersen’s study results suggest that overweight-specific survey instruments are more sensitive in detecting changes within QoL. Notably, all studies included in this systematic review, except for Kraschnewski et al., used generic survey instruments, which may also have been a reason for why no statistically significant findings could be identified.
6. To assess the effects achieved according to QoL, their clinical relevance for the patients themselves must be considered in addition to their statistical significance. When selecting suitable operationalization instruments, care should therefore already be taken to ensure that a minimal clinically important difference (MCID) is available for the respective instrument in the context of the specific indication [49]. The term “MCID” refers to “the smallest difference in score in the domain of interest which the patient perceives as beneficial and which would mandate, in the absence of troublesome side-effects and excessive cost, a change in the patient’s management” [50] and is therefore a central measure in clinical research because it helps to evaluate the significance of changes that are clinically relevant from the perspective of patients and physicians [51].
7. Except for two studies, all studies included in this systematic review have been published within the last five years and conducted in Europe. A study by Haraldstad et al. also highlighted that research on QoL is predominantly undertaken in industrialized countries. It is crucial to note that the understanding of the QoL varies across different cultures [16].
8. The study participants, on average, ranged between 45 and 57 years old. Age has been shown to influence various domains of QoL differently; for instance, physical functioning tends to become more impaired with age, while domains such as self-esteem and public discomfort may improve [52].
9. The average BMI of the included participants, excluding one study, was over 30 kg/m^2^, indicating obesity. Studies demonstrate a causal relationship between increasing obesity and low QoL, particularly in individuals with class III obesity (BMI ≥ 40 kg/m^2^) [53].
10. Only two studies indicate a statistically significant difference between IG and CG according to behavior change endpoints, which could be another explanation for the lack of statistically significant improvements in QoL (and BMI). Three studies did not assess any endpoints in relation to a possible change in behavior. Possible changes in QoL should always be considered in relation to behavioral changes that have occurred.
11. Adherence data was reported in four of the included studies. These data are important to be able to assess and interpret the effects achieved in relation to QoL [54]. For this reason, the new Digital Act also recommends an application-accompanying performance measurement in which—in addition to patient satisfaction—the duration and intensity of app usage are also measured [55].

**Fig. 2:**
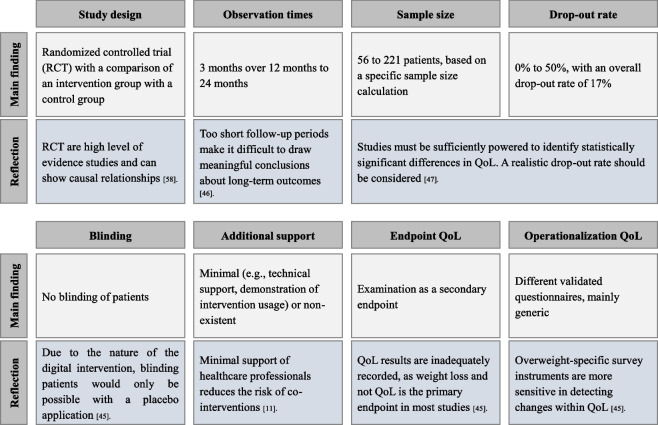
Reflection on main findings

Based on the limited and inconclusive main findings of our systematic review, additional evidence was used to reflect on design characteristics for the conception of future comparative DiHA studies (Fig. 2).

### Comparison with prior work

The current scientific evidence focuses on telemedicine interventions, technology-based interventions, ehealth interventions or wearable and smartphone applications, primarily to improve weight management in patients with overweight and/or obesity. However, none of the studies conducted to date have focused on proving the effectiveness of the specific DiHA on QoL of those patients. Our systematic review closes this research gap.

In a systematic review, Knapp et al. found frequently addressed patient-reported outcome measures in assessing telemedicine interventions, deeming QoL one of the most important outcomes [19]. Raaijmakers et al. aimed to investigate the effectiveness of technology-based interventions on weight and QoL in patients with overweight or obesity through a systematic review (*n* = 27). The interventions examined in the studies incorporated elements such as group sessions, meetings, face-to-face information sessions, or telephone calls, introducing the challenge of potential co-interventions. Only two studies within the review analyzed QoL; however, no significant change in QoL was identified in these studies, and the reporting of QoL outcomes was limited [23]. Wright et al. aimed to investigate the effectiveness of eHealth interventions for adults who are scheduled for or having undergone bariatric surgery through a systematic review (*n* = 14). The interventions assessed in the studies involved components such as videoconferences or were delivered by professionals like nurse managers, psychometrists, or psychologists, introducing a notable risk of co-interventions. Among the included studies, five measured QoL, and three demonstrated a significant improvement in QoL. It is worth noting that one of these studies was a pilot study, and another explored a videoconferencing based psychoeducational group intervention, which does not align with the characteristics of a DiHA [56]. Dehghan Ghahfarokhi et al. conducted a meta-analysis (*n* = 26) to explore the effectiveness of wearable and smartphone applications on physical activity, QoL, and cardiovascular health outcomes in overweight or obese adults. In contrast to the present systematic review, where no significant pooled SMD between the IG and CG in QoL was identified, Dehghan Ghahfarokhi et al. found a noteworthy SMD (SMD = 0.33; *p* < 0.001) [20].

### Strengths and limitations

Owing to the limited number of included studies, evidence-based recommendations for DiHA on whether and how DiHA may enhance QoL in patients with overweight or obesity proves challenging. The restricted number of included studies stems from the precise definition of eligibility criteria, an imperative step in addressing the research questions:

1. The interventions and their functionalities had to align with the specific criteria for a DiHA, resulting in numerous studies being excluded. A central requirement for DiHA classification is its primarily use by patients with direct interaction with the application. Collaborative interaction between patients and healthcare professionals becomes viable only when the positive healthcare effect is attributed to the digital technology itself, rather than the interaction with the healthcare professional. The autonomy of a digital intervention, operating independent of a healthcare professional, holds particular significance, especially in situations where prolonged treatment waiting times exist or when certain patient groups encounter barriers to accessing the healthcare system. The precision of this intervention definition serves as a strength by minimizing the risk of co-interventions, as the influence of a healthcare professional was either non-existent or minimal in all studies.
2. To establish a positive healthcare effect of a DiHA, at least one scientific, comparative study is required, and to date, all DiHA manufacturers have conducted an RCT [57]. Consequently, only studies with a CG as well as (randomized) controlled studies and cross-over studies were included. While this approach excludes potentially relevant comparative studies, it ensures a high level of evidence inherent in RCT and the capacity to demonstrate casual relationships [58]. It is essential to acknowledge that the lack of blinding of patients in all studies might have influenced the effects achieved, given the patients’ awareness of the intervention. However, due to the inherent nature of a digital intervention, blinding patients would only be feasible with a placebo application. For this reason, most of the DiHA studies already conducted achieve a high risk of bias [59], which must be considered when interpreting the risk of bias in this systematic review. An additional limitation is the constraint of study inclusion to the German or English language. Furthermore, the generalizability of the results is circumscribed, considering variations in the healthcare systems under examination and the age restriction imposed on the study population in all included studies. Due to the focus of the study, which was very specific, other potentially relevant outcomes such as diet or physical activity, which may have changed because of using the DiHA, were not considered.

This systematic review employed rigorous and transparent methods to identify the best available evidence. Following established guidelines for systematic reviews, specifically the PRISMA framework ensured a systematic and comprehensive approach. The results were reported in accordance with the CONSORT-EHEALTH checklist. Methodological procedures, including title-abstract screening, full-text screening, and quality assessment were concurrently conducted by two researchers independently, minimizing the likelihood of overlooking relevant literature or erroneously including studies that did not meet the specified eligibility criteria. Additionally, an update of the research was performed in November 2023, ensuring the inclusion of the latest evidence.

### Future research

Digital interventions hold the potential to support the treatment of overweight and obesity; however, additional findings are essential to comprehensively understand the impact of DiHA on the QoL of patients with overweight and obesity. A necessity exists for more randomized, controlled, and high-quality studies to assess the impact of DiHA on QoL in patients with overweight or obesity in this context. As gender, age and baseline BMI are important predictors of QoL, future studies should be large enough to stratify the results according to these variables. Moreover, future studies should delve into understanding whether the improvements in QoL is attributed to the weight loss itself, the digital health app, or another variable. Exploring the threshold of weight loss facilitated by a digital health app that leads to enhanced QoL would also be a valuable avenue for research.

## Conclusions

This systematic review offers an insight into the effectiveness of DiHA on QoL in patients with overweight or obesity. Notably, none of the examined interventions demonstrated a statistically significant impact on QoL. The identified heterogeneity in study durations, sample sizes, and particularly in the operationalization of QoL call for standardized digital health trials. Future randomized, controlled, and high-quality studies are required to comprehensively assess the impact of DiHA on QoL in patients with overweight or obesity. Our findings help to develop DiHA with focus on improving patient’s QoL and to decide on characteristics for the design of future DiHA studies.

## Supplementary Information

Additional file 1. Individual search strategies within the selected databases.

Additional file 2. Overview of the excluded studies.

Additional file 3. Study characteristics of the included studies.

Additional file 4. Quality assessment of the included studies.

Additional file 5. Study results on BMI and weight.

## Data Availability

Data from the following studies were included in this systematic review: Mensorio MS, Cebolla-Martí A, Rodilla E, Palomar G, Lisón JF, Botella C, et al. Analysis of the efficacy of an internet-based self-administered intervention ("Living Better") to promote healthy habits in a popula-tion with obesity and hypertension: An exploratory randomized controlled trial. Int J Med Inform. 2019; 124:13–23. Epub 2018/12/21. doi: 10.1016/j.ijmedinf.2018.12.007 PMID: 30784422 .Mangieri CW, Johnson RJ, Sweeney LB, Choi YU, Wood JC. Mobile health applications enhance weight loss efficacy following bariatric surgery. Obes Res Clin Pract. 2019; 13:1–4. Epub 2019/02/28. doi: 10.1016/j.orcp.2019.01.004 PMID: 30826256 .Kraschnewski JL, Stuckey HL, Rovniak LS, Lehman EB, Reddy M, Poger JM, et al. Efficacy of a weight-loss website based on positive deviance. A randomized trial. Am J Prev Med. 2011; 41:610–4. doi: 10.1016/j.amepre.2011.08.012 PMID: 22099238 .McConnon A, Kirk SF, Cockroft JE, Harvey EL, Greenwood DC, Thomas JD, et al. The Internet for weight control in an obese sample: results of a randomised controlled trial. BMC Health Serv Res. 2007; 7:1–9. Epub 2007/12/19. doi: 10.1186/1472-6963-7-206 PMID: 18093289 .Roth L, Ordnung M, Forkmann K, Mehl N, Horstmann A. A randomized-controlled trial to evaluate the app-based multimodal weight loss program zanadio for patients with obesity. Obesity (Silver Spring). 2023; 31:1300–10. doi: 10.1002/oby.23744 PMID: 37140392 .Múzquiz-Barberá P, Ruiz-Cortés M, Herrero R, Vara MD, Escrivá-Martínez T, Baños RM, et al. "Own doc-tor" presence in a web-based lifestyle intervention for adults with obesity and hyperten-sion: A random-ized controlled trial. Front Public Health. 2023; 11:1115711. Epub 2023/03/14. doi: 10.3389/fpubh.2023.1115711 PMID: 36998287 .Gemesi K, Winkler S, Schmidt-Tesch S, Schederecker F, Hauner H, Holzapfel C. Efficacy of an app-based multimodal lifestyle intervention on body weight in persons with obesity: results from a ran-domized controlled trial. Int J Obes (Lond). 2023. Epub 2023/11/28. doi: 10.1038/s41366-023-01415-0 PMID: 38017117 .

## Abbreviations

ANCOVA: Analysis of covariance
ANOVA: Analysis of variance
CG: Control group
CI: Confidence interval
COS: Core outcome set
DiHA: Digital health application
E66.00: Obesity due to excessive calorie intake: Obesity grade I (WHO) in patients 18 years and older
E66.01: Obesity due to excessive calorie intake: Obesity grade II (WHO) in patients 18 years and older
EBL: Excess BMI loss
EOSS: Edmonton Obesity Staging System
EWL: Excess bodyweight loss
f: female
ICD-10: International Statistical Classification of Diseases and Related Health Problems
IG: Intervention group
IQR: Interquartile range
IQWiG: Institute for Quality and Efficiency in Health Care
ITT: Intention-to-treat
IWQOL-Lite: Impact of Weight on Quality of Life-Lite
LOCF: Last-Observation-Carried-Forward
m: male
M: mean
MCID: Minimal clinically important difference
MD: Mean difference
n: absolute frequency
n.a.: not applicable
n.p.: not possible
n.r.: not reported
PRISMA: Preferred Reporting Items for Systematic Reviews and Meta-Analyses
PROSPERO: Prospective Register of Systematic Reviews
QoL: Quality of life
RCT: Randomized contolled trial
RoB2: Risk of Bias Tool version 2
SD: Standard deviation
SF-36: Short-Form-36
SMD: Standard mean difference
WHO: World Health Organization
