# Supplementary material for: Effectiveness of digital health applications on the quality of life in patients with overweight or obesity: a systematic review
Source: Arch Public Health. 2025 Jan 9;83:3. doi: 10.1186/s13690-024-01474-3 (PMC11715991; doi:10.1186/s13690-024-01474-3)
Supplement: Supplementary file 1 — Additional file 1. Individual search strategies within the selected databases. [file 13690_2024_1474_MOESM1_ESM.docx]

Additional file 1: Individual search strategies within the selected databases

Database search strategy for PubMed (performed on 21/03/2023)

| PICO(S)-Criteria | No. | Search term | Results |
| --- | --- | --- | --- |
| Population | #1 | Adiposity[Mesh] OR adipos*[tiab] OR corpulenc*[all fields] OR "excess body weight"[all fields] OR "fat overload syndrome"[all fields] OR Obesity[Mesh] OR obes*[tiab] OR Overnutrition[Mesh] OR overnutrition[all fields] OR Overweight[Mesh] OR overweight[tiab] OR over-weight[tiab] OR "over weight"[tiab] OR overeating[all fields] OR over-eating[all fields] OR "over eating" [all fields] | 554,247 |
| Intervention | #2 | app[tiab] OR "app-assisted"[all fields] OR Cell Phone[Mesh] OR cellphone*[tiab] OR "cellular phone*"[all fields] OR digital[tiab] OR "digital health"[all fields] OR "digital health application*"[all fields] OR DiGA[all fields] OR DiHA[all fields] OR Digital Technology[Mesh] OR "digital technolog*"[tiab] OR Ehealth[tiab] OR e-health[tiab] OR "electronic health"[tiab] OR emedicine*[all fields] OR e-medicine*[all fields] OR "e medicine*"[all fields] OR "electronic medicine*"[all fields] OR "e therapy"[all fields] OR "e therap*"[all fields] OR etherap*[all fields] OR e-therap*[all fields] OR Electronic Mail[Mesh] OR e-mail*[tiab] OR "electronic mail"[tiab] OR "electronic messaging"[all fields] OR e-learning[all fields] OR "electronic learning"[all fields] OR "home monitoring"[all fields] OR home-monitor*[all fields] OR mhealth[tiab] OR m-health[all fields] OR "mobile health"[tiab] OR Mobile Applications[Mesh] OR "mobile application*"[tiab] OR "mobile health application*"[tiab] OR "mobile app*"[tiab] OR mobilephone*[tiab] OR "mobile phone*"[tiab] OR "mobile technolog*"[tiab] OR phone*[tiab] OR "portable electronic*"[all fields] OR "simulation tool*"[all fields] OR SMS[tiab] OR "short message*"[tiab] OR "short messaging"[tiab] OR Smartphone[Mesh] OR smartphone*[tiab] OR "smart phone*"[tiab] OR "smartphone app*"[tiab] OR Social Networking[Mesh] OR "social network*"[tiab] OR telemedicine[Mesh] OR Telemedicine[tiab] OR telemed*[tiab] OR telehealth[tiab] OR Telematic*[all fields] OR teletherap*[all fields] OR telemonitor*[all fields] OR tele-monitor*[all fields] OR Text Messaging[Mesh] OR "text messag*"[tiab] OR texting[all fields] OR virtual[tiab] OR Virtual Reality[Mesh] OR "virtual reality"[tiab] OR web-based[tiab] OR "web based"[tiab] OR wearable*[tiab] | 560,984 |
| Outcome | #3 | Quality of Life[Mesh] OR "quality of life"[tiab] OR "life quality"[tiab] OR "health-related quality of life"[tiab] OR "health related quality of life"[tiab] OR HRQOL[tiab] OR QoL[tiab] OR QL[tiab] | 429,151 |
|  | #4 | #1 AND #2 AND #3 | 421 |

Database search strategy for Cochrane Library (performed on 21/03/2023)

| PICO(S)-Criteria | No. | Search term | Results |
| --- | --- | --- | --- |
| Population | #1 | MeSH descriptor: [Adiposity] explode all trees | 956 |
|  | #2 | (adipos*):ti,ab,kw | 9,255 |
|  | #3 | corpulenc* | 9 |
|  | #4 | "excess body weight" | 208 |
|  | #5 | "fat overload syndrome" | 10 |
|  | #6 | MeSH descriptor: [Obesity] explode all trees | 18,204 |
|  | #7 | (obes*):ti,ab,kw | 51,777 |
|  | #8 | MeSH descriptor: [Overnutrition] explode all trees | 21,541 |
|  | #9 | overnutrition | 234 |
|  | #10 | MeSH descriptor: [Overweight] explode all trees | 21,526 |
|  | #11 | (overweight):ti,ab,kw | 20,140 |
|  | #12 | (over-weight):ti,ab,kw | 175 |
|  | #13 | overeating | 450 |
|  | #14 | over-eating | 89 |
|  | #15 | "over eating" | 89 |
|  | #16 | #1 OR #2 OR #3 OR #4 OR #5 OR #6 OR #7 OR #8 OR #9 OR #10 OR #11 OR #12 OR #13 OR #14 OR #15 | 60,059 |
| Intervention | #17 | (app):ti,ab,kw | 8,032 |
|  | #18 | "app-assisted" | 29 |
|  | #19 | MeSH descriptor: [Cell Phone] explode all trees | 2,890 |
|  | #20 | ("cellphone*"):ti,ab,kw | 153 |
|  | #21 | "cellular phone*" | 180 |
|  | #22 | MeSH descriptor: [Digital Technology] explode all trees | 14 |
|  | #23 | (digital):ti,ab,kw | 16,620 |
|  | #24 | "digital health" | 6,580 |
|  | #25 | "digital health application*" | 19 |
|  | #26 | DiGA | 12 |
|  | #27 | DiHA | 0 |
|  | #28 | (Ehealth):ti,ab,kw | 1,387 |
|  | #29 | (e-health):ti,ab,kw | 1,410 |
|  | #30 | ("electronic health"):ti,ab,kw | 2,829 |
|  | #31 | emedicine* | 12 |
|  | #32 | e-medicine* | 17 |
|  | #33 | "e medicine*" | 834 |
|  | #34 | "electronic medicine*" | 10 |
|  | #35 | "e therapy" | 1,141 |
|  | #36 | "e therapies" | 404 |
|  | #37 | etherap* | 51 |
|  | #38 | (e-therap*):ti,ab,kw | 914 |
|  | #39 | MeSH descriptor: [Electronic Mail] explode all trees | 406 |
|  | #40 | (e-mail*):ti,ab,kw | 2,831 |
|  | #41 | ("electronic mail"):ti,ab,kw | 453 |
|  | #42 | "electronic messaging" | 47 |
|  | #43 | e-learning | 1,099 |
|  | #44 | "electronic learning" | 69 |
|  | #45 | "home monitoring" | 732 |
|  | #46 | home-monitor* | 814 |
|  | #47 | (mhealth):ti,ab,kw | 1,984 |
|  | #48 | m-health | 2,414 |
|  | #49 | ("mobile health"):ti,ab,kw | 1,836 |
|  | #50 | MeSH descriptor: [Mobile Applications] explode all trees | 1,506 |
|  | #51 | ("mobile application*"):ti,ab,kw | 2,705 |
|  | #52 | ("mobile health application*"):ti,ab,kw | 166 |
|  | #53 | ("mobile app*"):ti,ab,kw | 1,289 |
|  | #54 | (mobilephone*):ti,ab,kw | 104 |
|  | #55 | ("mobile phone*"):ti,ab,kw | 3,488 |
|  | #56 | ("mobile technolog*"):ti,ab,kw | 0 |
|  | #57 | (phone*):ti,ab,kw | 19,274 |
|  | #58 | "portable electronic*" | 66 |
|  | #59 | "simulation tool*" | 30 |
|  | #60 | (SMS):ti,ab,kw | 2,686 |
|  | #61 | ("short message*"):ti,ab,kw | 841 |
|  | #62 | ("short messaging"):ti,ab,kw | 117 |
|  | #63 | MeSH descriptor: [Smartphone] explode all trees | 902 |
|  | #64 | (smartphone*):ti,ab,kw | 6,555 |
|  | #65 | ("smart phone*"):ti,ab,kw | 862 |
|  | #66 | ("smartphone app*"):ti,ab,kw | 1,087 |
|  | #67 | ("smart phone app*"):ti,ab,kw | 60 |
|  | #68 | MeSH descriptor: [Social Networking] explode all trees | 187 |
|  | #69 | ("social network*"):ti,ab,kw | 1,422 |
|  | #70 | MeSH descriptor: [Telemedicine] explode all trees | 4,006 |
|  | #71 | (telemedicine):ti,ab,kw | 5,451 |
|  | #72 | (telemed*):ti,ab,kw | 5,575 |
|  | #73 | (telehealth):ti,ab,kw | 3,177 |
|  | #74 | telematic* | 124 |
|  | #75 | teletherap* | 295 |
|  | #76 | telemonitor* | 1,361 |
|  | #77 | tele-monitor* | 143 |
|  | #78 | MeSH descriptor: [Text messaging] explode all trees | 1,379 |
|  | #79 | "text messag*" | 137 |
|  | #80 | "texting" | 468 |
|  | #81 | (virtual):ti,ab,kw | 10,401 |
|  | #82 | MeSH descriptor: [Virtual Reality] explode all trees | 784 |
|  | #83 | ("virtual reality"):ti,ab,kw | 5,406 |
|  | #84 | (web-based):ti,ab,kw | 9,238 |
|  | #85 | ("web based"):ti,ab,kw | 9,238 |
|  | #86 | MeSH descriptor: [Wearable Electronic Devices] explode all trees | 814 |
|  | #87 | ("wearable computer"):ti,ab,kw | 17 |
|  | #88 | (wearable*):ti,ab,kw | 2,045 |
|  | #89 | #17 OR #18 OR #19 OR #20 OR #21 OR #22 OR #23 OR #24 OR #25 OR #26 OR #27 OR #28 OR #29 OR #30 OR #31 OR #32 OR #33 OR #34 OR #35 OR #36 OR #37 OR #38 OR #39 OR #40 OR #41 OR #42 OR #43 OR #44 OR #45 OR #46 OR #47 OR #48 OR #49 OR #50 OR #51 OR #52 OR #53 OR #54 OR #55 OR #56 OR #57 OR #58 OR #59 OR #60 OR #61 OR #62 OR #63 OR #64 OR #65 OR #66 OR #67 OR #68 OR #69 OR #70 OR #71 OR #72 OR #73 OR #74 OR #75 OR #76 OR #77 OR #78 OR #79 OR #80 OR #81 OR #82 OR #83 OR #84 OR #85 OR #86 OR #87 OR #88 O | 80,620 |
| Outcome | #90 | MeSH descriptor: [Quality of Life] explode all trees | 35,347 |
|  | #91 | ("quality of life"):ti,ab,kw | 140,331 |
|  | #92 | ("life quality"):ti,ab,kw | 5,340 |
|  | #93 | ("health-related quality of life"):ti,ab,kw | 21,262 |
|  | #94 | ("health related quality of life"):ti,ab,kw | 21,262 |
|  | #95 | HRQOL:ti,ab,kw | 7,046 |
|  | #96 | QoL:ti,ab,kw | 25,439 |
|  | #97 | QL:ti,ab,kw | 1,280 |
|  | #98 | #91 OR #92 OR #93 OR #94 OR #95 OR #96 OR #97 | 146,769 |
|  | #99 | #16 AND #89 AND #98 | 170 |
| Filter: Trials |  |  | 110 |

Database search strategy for Embase (performed on 21/03/2023)

| PICO(S)-Criteria | No. | Search term | Results |
| --- | --- | --- | --- |
| Population | #1 | adipos*.ab. or adipos*.ti. or corpulenc*.af. or "excess body weight".af. or "fat overload syndrome".af. or exp obesity/ or obes*.ab. or obes*.ti. or exp overnutrition/ or overnutrition.af. or overweight.ab. or overweight.ti. or over-weight.ab or over-weight.ti or "over weight".ab or "over weight".ti or overeating.af or over-eating.af or "over eating".af | 864,616 |
| Intervention | #2 | app.ab. or app.ti. or "app-assisted".af. or "cell phone*".af. or cellphone*.ab. or cellphone*.ti. or "cellular phone*".af. or digital.ab. or digital.ti. or "digital health".af. or "digital health application*".af. or DiGA.af. or DiHA.af. or exp digital technology/ or "digital technology".ab. or "digital technology".ti. or ehealth.ab. or ehealth.ti. or e-health.ab. or e-health.ti. or "electronic health".ab. or "electronic health".ti. or emedicine*.af. or e-medicine*.af. or "e medicine*".af. or "electronic medicine*".af. or "e therapy".af. or "e therap*".af. or etherap*.af. or e-therap*.ab. or e-therap*.ti. or "electronic mail".ab. or "electronic mail".ti. or exp e-mail/ or e-mail.ab. or e-mail.ti or "electronic messaging".af. or exp e-learning/ or e-learning.af. or "electronic learning".af. or exp home monitoring/ or "home monitoring".af. or home-monitor*.af. or mhealth.ab. or mhealth.ti. or m-health.af. or "mobile health".ab. or "mobile health".ti. or exp mobile application/ or "mobile application*".ab. or "mobile application*".ti. or exp mobile health application/ or "mobile health application*".ab. or "mobile health application*".ti. or "mobile app".ab. or exp mobile phone/ or mobilephone*.ab. or mobilephone*.ti. or "mobile phone*".ab. or "mobile phone*".ti. or "mobile technolog*".ab. or "mobile technolog*".ti. or phone*.ab. or phone*.ti. or "portable electronic*".af. or "simulation tool".af. or SMS.ab. or SMS.ti. or "short message".ab. or "short message".ti. or "short messaging".ab. or "short messaging".ti. or exp smartphone/ or smartphone*.ab. or smartphone*.ti. or "smart phone*".ab. or "smart phone*".ti. or "smartphone app*".ab. or "smartphone app*".ti. or exp social network/ or "social network*".ab. or "social network*".ti. or exp telemedicine/ or telemedicine.ab. or telemedicine.ti. or telemed*.ab. or telemed*.ti. or exp telehealth/ or telehealth.ab. or telehealth.ti. or telematic*.af. or teletherap*.af. or telemonitor*.af. or tele-monitor*.af. or exp text messaging/ or "text messag*".ab. or "text messag*".ti. or texting.af. or virtual.ab. or virtual.ti. or exp virtual reality/ or "virtual reality".ab. or "virtual reality".ti. or web-based.ab. or web-based.ti. or "web based".ab. or "web based".ti. or exp wearable computer/ or "wearable computer".ab. or "wearable computer".ti. or wearable*.ab. or wearable*.ti. | 759,787 |
| Outcome | #3 | exp "quality of life"/ or "quality of life".ab. or "quality of life".ti. or "life quality".ab. or "life quality".ti. or "health-related quality of life".ab. or "health-related quality of life".ti. or "health related quality of life".ab. or "health related quality of life".ti. or HRQOL.ab. or HRQOL.ti. or QoL.ab. or QoL.ti. or QL.ab. or QL.ti. | 777,364 |
|  | #4 | #1 AND #2 AND #3 | 1064 |
