# Supplementary material for: Effectiveness of digital health applications on the quality of life in patients with overweight or obesity: a systematic review
Source: Arch Public Health. 2025 Jan 9;83:3. doi: 10.1186/s13690-024-01474-3 (PMC11715991; doi:10.1186/s13690-024-01474-3)
Supplement: Supplementary file 2 — Additional file 2. Overview of the excluded studies. [file 13690_2024_1474_MOESM2_ESM.docx]

**Additional file 2: Overview of the excluded studies**

Overview of excluded studies incl. reasons for exclusion

| Author (Year) | Title | Reason for exclusion |
| --- | --- | --- |
| Records identified through database searching | | |
| (1) De Niet et al. (2012) | The effect of a short message service maintenance treatment on body mass index and psychological well-being in overweight and obese children: a randomized controlled trial | I: "The feedback was checked for plausibility by a researcher who tailored the feedback message when needed." (p. 208) |
| (2) Patrick et al. (2013) | Outcomes of a 12-month technology-based intervention to promote weight loss in adolescents at risk for type 2 diabetes | I: Website only: "The purpose of the weekly emails was to remind the participants to complete the web tutorials. If participants did not log on to the web program, they received repeated reminders via email and, if necessary, a phone call from a health counselor." (p. 762)  Website + group session: "Participants in this condition also received brief (~20 min) bimonthly phone calls from the health counselor reviewing concepts presented in the web tutorial and reinforcing behavioral strategies such as goal setting and problem solving of barriers/solutions." (p. 763)  Website + SMS: "Participants could also  communicate via text messages with a health counselor if they had questions. Participants were provided with cellphones and prepaid text message plans that allowed research staff to monitor SMS use." (p. 763)" |
| (3) Postrach et al. (2012) | Efficacy of an internet-based weight loss program: A proof-of-principle trial | S: Conference Abstract |
| (4) Lee et al. (2020) | The Effect of Social Norm-based Intervention with Observable Behaviour on Physical Activity among Adolescents: A Randomized Controlled Trial | I: "For participants in the Anonymous Arm, the weekly information included step count ranked from the highest to lowest within all participants in the same group. Participants in the Onymous Arm receive the same information plus the (real) full names of the participants next to the step count." (p. 3) |
| (5) Likhitweerawong et al. (2021) | Effectiveness of mobile application on changing weight, healthy eating habits, and quality of life in children and adolescents with obesity: a randomized controlled trial | I: "Messaging from the healthcare provider was sent similarly to the users every two weeks to remind and motivate them to keep up the intervention." (Additional file 1) |
| (6) Morgan et al. (2013) | The SHED-IT Community Trial: A randomised controlled trial of Internet- and paper-based weight loss programs tailored for overweight and obese men | I: "Over the course of the three months, each participant was emailed seven individualized feedback sheets by research assistants. Online diaries were reviewed weekly in the first month, fortnightly in the second month, and once in the third month." (p. 142) |
| (7) Morgan et al. (2012) | Physical activity outcomes from the SHED-IT RCT: An evaluation of theoretically-based, gender-sensitised weight loss programs for men | S: Conference Abstract |
| (8) Thomas et al. (2019) | Comparison of Smartphone-Based Behavioral Obesity Treatment With Gold Standard Group Treatment and Control: A Randomized Trial | O: not QoL |
| (9) Taylor et al. (2020) | Adding web-based behavioural support to exercise referral schemes for inactive adults with chronic health conditions: the e-coachER RCT | P: one or more of the following conditions:  -obesity [i.e. a body mass index (BMI) of 30–40 kg/m2]  -diagnosis of hypertension  -prediabetes  -type 2 diabetes  -lower limb osteoarthritis  -current or recent history of treatment for depression  -categorised as inactive (p.13)  P: BMI range: 18.8–40.5 (p.27) |
| (10) Rhein et al. (2018) | Web-based educational health as an adjuvant tool on interdisciplinary behavioral therapy, improving the food habits and body image in obese women | S: Conference Abstract |
| (11) Roth et al. (2022) | The evaluation of zanadio - a digital health application for people with obesity | S: Conference Abstract |
| (12) Stewart et al. (2015) | A mobile health driven walking program improves quality of life but not fitness or fatness in obese sedentary women | S: Conference Abstract |
| (13) Stasinaki et al. (2018) | A novel digital health intervention improves physical performance in obese youth | S: Conference Abstract |
| (14) Struckmeyer et al. (2022) | Significant improvement in dietary behaviors and quality of life among adolescents with obesity in the COVID19 lockdown through telehealth | S: Conference Abstract |
| (15) Sousa et al. (2015) | Controlled trial of an Internet-based intervention for overweight teens (Next.Step): effectiveness analysis | I: "In addition to the platform manager (nurse), the program also had the direct support of an interdisciplinary team (including paediatrician, nutritionist, exercise physiologist and psychologist) who intervened when requested by the case manager." (p. 1146) |
| (16) Chen et al. (2019) | Smartphone-based Healthy Weight Management Intervention for Chinese American Adolescents: Short-term Efficacy and Factors Associated With Decreased Weight | S: Pilot Study |
| (17) Bughin et al. (2021) | Impact of a Mobile Telerehabilitation Solution on Metabolic Health Outcomes and Rehabilitation Adherence in Patients With Obesity: Randomized Controlled Trial | I: "Patients with TRG had 2 teleconsultations at 1 and 2 months." (p.3) |
| (18) Donelly et al. (2007) | Comparison of a phone vs clinic approach to achieve 10% weight loss | I: "The phone group participated via a group conference call with the health educator" (p. 1271) |
| (19) Collins et al. (2010) | Evaluation of a commercial web-based weight loss and weight loss maintenance program in overweight and obese adults: a randomized controlled trial | S: Study Protocol |
| (20) Dabbas et al. (2019) | Use of new technologies for the follow-up in adolescent obesity; mobile health intervention (MHI) a randomized controlled trial | S: Conference Abstract |
| (21) Manzoni et al. (2016) | Assessment of body image perception in patients with severe obesity using virtual reality | I: "After the first inpatients week, participants entered five weekly group sessions similar to the CBT ones (focused on concerns about body weight and shape and problematic eating) and 10 biweekly VR sessions." (p. 136) |
| (22) Hoerster et al. (2022) | Effect of a Remotely Delivered Self-directed Behavioral Intervention on Body Weight and Physical Health Status Among Adults With Obesity: The D-ELITE Randomized Clinical Trial | I: "Coach contact was minimal, aside from  a 1-time orientation by telephone to review materials and set initial goals, biweekly standardized reminders, and as needed messages." (p. 2231) |
| (23) Kraschnewski et al. (2010) | A web-based intervention for weight loss: Disseminating effective habits of successful weight losers (positive deviants) | S: Conference Abstract |
| (24) Keating et al. (2019) | Text messaging as an intervention for weight loss in emerging adults | O: not QoL |
| (25) Fonseca et al. (2015) | The Next.Step program: Promoting health among overweight adolescents trough technological interfaces | S: Conference Abstract |
| (26) Fonseca et al. (2016) | Effectiveness analysis of an internet-based intervention for overweight adolescents: Next steps for researchers and clinicians | I: "The intervention length was 12 weeks and was based on a case management methodology. So, the program had the direct support of an interdisciplinary team (including paediatrician, nutritionist, exercise physiologist, and psychologist) who intervened when requested by the case manager (nurse)." (p.3) |
| (27) Fichtner et al. (2022) | Evaluation of an Interactive Web-Based Health Program for Weight Loss-A Randomized Controlled Trial | P: BMI < 25=13.5% (no subgroup analysis) |
| (28) Sysko et al. (2022) | An Initial Test of the Efficacy of a Digital Health Intervention for Bariatric Surgery Candidates | I: "Coaches help each user identify individual goals; focus on diet changes, exercise, and wellness; troubleshoot weight loss barriers; individualize feedback; find ways to adapt to lifestyle changes; and increase motivation. Coaches schedule weekly check-ins with users to review weight loss and review overall progress." (p. 3642) |
| (29) Lauti et al. (2018) | A Randomised Trial of Text Message Support for Reducing Weight Regain Following Sleeve Gastrectomy | I: "Participants were randomised to TMS or usual care. TMS consisted of a daily text message delivered each morning for a period of 1 year together with usual care." (p. 2) |
| (30) Kurscheid et al. (2019) | App-controlled feedback devices can support sustainability of weight loss. Multicentre QUANT-study shows additional weight loss and gain of QoL via multiple feedback-devices in OPTIFAST®52-program | I: applications that only read other devices and transmit data^a^ |
| Additional records identified through other sources | | |
| (31) Collins et al. (2012) | A 12-Week Commercial Web-Based Weight-Loss Program for Overweight and Obese Adults: Randomized Controlled Trial Comparing Basic Versus Enhanced Features | I: "The Web-based program was underpinned by social cognitive theory and targeted key mediators of behavior change, including self-efficacy, goal setting, and self-monitoring of weight, body measurements, exercise, and diet; outcome expectations (knowledge-based Web components); modeling (interactive website features and demonstrations); and social support (forums, blogs, feedback, email, and telephone contact). " (p. 5) |
| (32) Lei et al. (2021) | Effectiveness of a Weight Loss Program Using Digital Health in Adolescents and Preadolescents | O: not QoL |
| (33) Womble et al. (2004) | A randomized controlled trial of a commercial internet weight loss program | I: "Participants met with a psychologist (L.G.W.) at baseline and at weeks 8, 16, 26, and 52 for 20 min/visit. " (p. 1012) |

^a^ Since this was not clear from the intervention description, Prof. Kurscheid was contacted to be able to define the character of the intervention: "the app only served to display the results on the cell phone, there was no further tip from the app."

I=intervention | O=outcome | P=population | S=study design
