# Supplementary material for: Effectiveness of digital health applications on the quality of life in patients with overweight or obesity: a systematic review
Source: Arch Public Health. 2025 Jan 9;83:3. doi: 10.1186/s13690-024-01474-3 (PMC11715991; doi:10.1186/s13690-024-01474-3)
Supplement: Supplementary file 3 — Additional file 3. Study characteristics of the included studies. [file 13690_2024_1474_MOESM3_ESM.docx]

**Additional file 3: Study characteristics of the included studies**

Study characteristic of Mensorio et al. (2019)

| Category | Description | | | |
| --- | --- | --- | --- | --- |
| Title | Analysis of the efficacy of an internet-based self-administered intervention ("Living Better") to promote healthy habits in a population with obesity and hypertension: An exploratory randomized controlled trial | | | |
| Inclusion Criteria | -age 18–65 years  -in clinical treatment for the prevention of metabolic syndrome or cardiac complications  -overweight or type I obesity (BMI > 25 and < 35 kg/m2) | | | |
| Exclusion Criteria | -no internet access  -taking more than three antihypertensive drugs  -diagnosis of diabetes; diagnosis of eating disorder  -having a disability that prevents or hinders exercise and physical activity  -receiving any treatment for weight loss elsewhere | | | |
| Gender (n (%)) | n.r. | | | |
| Age (M (SD)) | 53 (8.9); range=28–69 | | | |
| BMI (M (SD)) | IG=30.08 (2.65) | CG=30.15 (2.96) | | p=0.02 |
| Intervention | Online intervention "Living Better" + usual medical treatment:  -nine modules presented via web page  -3 pillars: (1) behavioral therapy techniques, (2) eating habits, (3) physical activity  -psychoeducation about healthy lifestyle and learning techniques; self-observation, self-instruction, behavioral recording, self-reinforcement, problem-solving, home-work | | | |
| Support | "Researchers will only contact participants if they stop accessing the modules for more than two weeks after they are posted. In this case, a reminder email will be sent. After three weeks without intervention access, a reminder telephone call will be made in order to ask the participants about any difficulties or doubts related to the use of the online protocol and help them solve any problems." (Banos et al. 2015, S. 5) | | | |
| Control | Treatment as usual + online intervention "Living Better" after 3 months  -routine medical examinations in which patients are monitored and provided with information on lifestyles and diet (usually one medical visit every 6-8 months on average) | | | |
| Endpoints and Operationalization | (1) BMI, (2) waist and hip perimeter (centimeters), (3) physical activity and sedentary behavior (IPAQ-short), (4) **quality of life (QLI),** (5) overall sense of efficacy (GSES-12), (6) styles of eating (DEBQ), (7) anxiety, depression, stress (DASS-21), (8) stages of self-determination (BREQ-2) | | | |
| Study design | RCT | | | |
| Randomization | 1:1 | | | |
| Stratification | none | | | |
| Blinding | n.r. | | | |
| Observation times | t0=baseline; t1=3 months; t2= 6 months (FU1), t3=12 months (FU2) | | | |
| Sample size | 106 (IG=55; CG=51) | | | |
| Drop-Out | t1=15 (IG=12; CG=3); t2=67 (IG=35; CG=32); t3=63 (IG=45; CG=18) | | | |
| Place and time of study | Spain | | n.r. | |

BMI=body-mass-index | BREQ=Behavioral Regulation in Exercise Questionnaire | CG=control group | DASS=Depression, Anxiety, Stress Scale | DEBQ=Dutch Eating Behaviours Questionnaire | FU=follow-up | GSES=Self-Efficacy Questionnaire General | IG=intervention group | IPAQ=International Physical Activity Questionnaire | n=quantity | n.r.=not reported | m=mean | QLI=Quality of Life Index | RCT=randomized controlled trial | SD=standard deviation

Study characteristic of Mangieri et al. (2019)

| Category | Description | | |
| --- | --- | --- | --- |
| Title | Mobile health applications enhance weight loss efficacy following bariatric surgery | | |
| Inclusion Criteria | -age 18-89 years  -patients who had undergone a laparoscopic sleeve gastrectomy (LSG) within 1 year of enrollment | | |
| Exclusion Criteria | -pregnant females  -subjects with immediate post-operative complications  -patients who were not fluent in English | | |
| Gender (n (%)) | f: IG=24 (84%); CG=26 (92%) | m: IG=4 (16%); CG=2 (8%) | p=0.54 |
| Age (M (SD)) | IG=52.5 (9.0) | CG=53 (10.6) | p=0.89 |
| BMI (M (SD)) | IG=35.34 (8.27) | CG=36.97 (6.91) | p=0.43 |
| Intervention | MyFitnessPal application + standard post-operative care  -nutrition diary  -comparison of the number of calories with previously set goals  -training diary for documenting training duration, intensity, and number of calories burned  -progress overview | | |
| Support | "When the research coordinator contacted patients in the mHealth group it was simply to either remind them to use the application or provide technical support for the application and they did not instruct, educate, or in any other way alter the patients’ behavioral patterns." (Mangieri et al. 2019, S. 2) | | |
| Control | self-monitoring journals + standard post-operative care | | |
| Endpoints and Operationalization | Primary:  (1) percent of excess bodyweight loss:  %EWL=((preoperative weight-current weight)/(preoperative weight-ideal weight))*100  (2) percent of excess BMI loss:  %EBL=((preoperative BMI-current BMI)/(preoperative BMI-25)*100  Secondary: **(1) quality of life (RAND-36)** | | |
| Study design | RCT | | |
| Randomization | 1:1 | | |
| Stratification | None | | |
| Blinding | n.r. | | |
| Observation times | t0=baseline; t1=12 months; t2=24 months | | |
| Sample size | 56 (IG=28; CG=28) | | |
| Drop-Out | 0 | | |
| Place of study | USA | | |
| Time of study | n.r. | | |

BMI=body-mass-index | CG=control group | EBL=excess BMI loss | EWL=excess bodyweight loss | f=female | IG=intervention group | m=male | M=mean | n=quantity | n.r.=not reported | RAND=research and development | RCT=randomized controlled trial | SD=standard deviation

Study characteristic of Kraschnewski et al. (2011)

| Category | Description | | |
| --- | --- | --- | --- |
| Title | Efficacy of a weight-loss website based on positive deviance. A randomized trial | | |
| Inclusion Criteria | -BMI 27-40 kg/m2  -age 21-65 years  -internet access at home or work  -ability to speak and read English  -access to a scale to measure body weight ability to safely engage in physical activity (measured by the Physical Activity Readiness Questionnaire) | | |
| Exclusion Criteria | -pregnancy  -planned or past weight-loss surgery  -weight loss ≥ 15 lbs in prior 6 months  -history of heart disease, stroke, diabetes, cancer, and major cognitive or psychiatric  impairment | | |
| Gender (n (%)) | f: IG=31 (62.0); CG=38 (77.6) | m: IG=19 (38); CG=12 (22.4) | 0.09 |
| Age (M (SD)) | IG=50.7 (10.5) | CG=49.9 (11.5) | 0.72 |
| BMI (M (SD)) | 32.7 (4.2) | 33.7 (4.2) | 0.26 |
| Intervention | AchieveTogether intervention:  -36 weight-control behaviors  -target body weight provided at initial log-in; at all log-ins, participants entered their weight, height, and frequency of using the 36 weight-loss practices in the past 7 days  -algorithms matched participants to 3 role models closest to them on gender, age, and target body weight  -participants prompted to build a weight-loss plan by selecting their preferred practices and setting weekly goals for practice use | | |
| Support | "All participants were shown in person how to use the AchieveTogether weight-loss website at the baseline visit." (Kraschnewski et al. 2011, S. 611) | | |
| Control | wait-list CG; website access after 12 weeks | | |
| Endpoints and Operationalization | Primary: (1) weight loss  Secondary: (1) blood pressure, (2) daily caloric intake, **(3) quality of life (IWQOL)**, (4) weight control behaviors (Weight Control Practices Questionnaire) | | |
| Study design | RCT | | |
| Randomization | 1:1 | | |
| Stratification | None | | |
| Blinding | n.r. | | |
| Observation times | t0=baseline, t1=12 weeks | | |
| Sample size | 100 (IG=50; CG=50) | | |
| Drop-Out | 12 (IG=7; CG=5) | | |
| Place of study | USA | | |
| Time of study | 2009-2010 | | |

BMI=body-mass-index | CG=control group | f=female | FU=follow-up | IG=intervention group | IWQOL=Impact of Weight on Quality of Life questionnaire | m=male | M=mean | n.r.=not reported | RCT=randomized controlled trial | SD=standard deviation

Study characteristics of Mc Connon et al. (2007)

| Category | Description | |
| --- | --- | --- |
| Title | The Internet for weight control in an obese sample: results of a randomised controlled trial | |
| Inclusion Criteria | -BMI ≥ 30 kg/m2  -age 18–65 years  -ability to access the Internet at least once per week  -ability to read and write in English | |
| Exclusion Criteria | none | |
| Gender (n (%)) | f=170 (77) | m=51 (23) |
| Age (M (SD)) | 45.8 (10.6) | |
| BMI (Median [IQR]) | IG=34.5 [31.8-38.5] | CG=34.4 [31.9-38.9] |
| Intervention | Website:  -dietary advice, physical activity advice, behaviour therapy  -enable patients to manage their own care  -personalised advice  -specific motivational statements  -details of progress (self-reported weight loss) | |
| Support | "Participants randomised into the Internet group were given a demonstration of the website and its services, along with a username and password to access the website and were asked to log on to the website at least once a week over the trial period. " (McConnon et al. 2007, S. 3) | |
| Control | Usual care:  -continue with their usual approach to weight loss  -printed information | |
| Endpoints and Operationalization | Primary: (1) weight, (2) BMI  Secondary: (1) lifestyle behaviours, **(2) quality of life (EuroQol)** | |
| Study design | RCT | |
| Randomization | 1:1 | |
| Stratification | Gender, age group, BMI category | |
| Blinding | None | |
| Observation times | t0=baseline, t1=6 months, t2=12 months | |
| Sample size | 221 (IG=111; CG=110) | |
| Drop-Out | t1: IG=42, CG=27; t2: IG=15, CG=6 | |
| Place of study | UK | |
| Time of study | n.r. | |

BMI=body-mass-index | CG=control group | EuroQol=European Quality of Life questionnaire | f=female | IG=intervention group | IQR=interquartile range | m=male | M=mean | n=quantity| n.r.=not reported | RCT=randomized controlled trial | SD=standard deviation

Study characteristics of Roth et al. (2023)

| Category | Description | |
| --- | --- | --- |
| Title | A randomized-controlled trial to evaluate the app-based multimodal weight loss program zanadio for patients with obesity | |
| Inclusion Criteria | -BMI: 30-40 kg/m2  -all genders  -age 18-65 years  -ICD-10 diagnosis E66.00 and E66.01; classification according to EOSS level 0-2  -ownership of a mobile device  -fluency in written and spoken German | |
| Exclusion Criteria | -patients in preparation for bariatric surgery or after bariatric surgery (gastric bypass, gastric reduction, gastric banding, or similar; past 3 years)  -advanced concomitant physical diseases (corresponding to EOSS level 3)  -acute untreated or unstable mental disorders  -current pregnancy or pregnancy planned within next 12 months  -presence of secondary forms of obesity (Cushing syndrome, Prader-Willi syndrome, hypogonadism, etc.)  -hypothyroidism if not treated with medication in advance  -lack of change resources  -physical limitations that do not allow moderate independent physical activity | |
| Gender (n (%)) | f: IG=69 (90.7); CG=67 (91.8) | m: IG=7 (9.2); CG=6 (8.2) |
| Age (M (SD)) | 43.4 (10.9) | |
| BMI (M (SD)) | 35.8 (3.2) |  |
| Intervention | Application "zanadio":  -multimodal approach including validated methods from behavioral science, exercise therapy, and nutrition  -different features: (1) knowledge transfer; (2) change; and (3) motivation and support | |
| Support | None | |
| Control | Usual care:  -could include self-initiated or externally initiated weight loss attempts | |
| Endpoints and Operationalization | Primary: (1) weight change (%)  Secondary: (1) body fat distribution, (2) well-being (WHO-5), **(3) quality of life** (WHOQOL-BREF) | |
| Study design | RCT | |
| Randomization | 1:1 | |
| Stratification | BMI, age | |
| Blinding | None | |
| Observation times | t0=baseline, t1=3 months, t2=6 months, t3=9 months, t4=12 months | |
| Sample size | 150 (IG=77, CG=73) | |
| Drop-Out | t4: IG=9; CG=6 | |
| Place of study | Germany | |
| Time of study | 01/2021-03/2022 | |

BMI=body-mass-index | CG=control group | EOSS=Edmonton Obesity Staging System | f=female | ICD-10=International Statistical Classification of Diseases and Related Health Problems | IG=intervention group | m=male | M=mean n=quantity | RCT=randomized controlled trial | SD=standard deviation | WHO-5= WHO-5 Well-Being Index | WHOQOL-BREF=World Health Organization Quality of Life-BREF

Study characteristics of Múzquiz-Barberá et al. (2023)

| Category | Description | |
| --- | --- | --- |
| Title | “Own doctor” presence in a web-based lifestyle intervention for adults with obesity and  hypertension: A randomized controlled trial | |
| Inclusion Criteria | -age 18-75 years  -patients with hypertension who were overweight (BMI >24.9 kg/m2 and <30 kg/m2) or who had type I obesity (BMI >29.9 kg/m2 and <35 kg/m2)  -patients that saw the same physician specialized in hypertension | |
| Exclusion Criteria | -patients who had not come for at least 1 visit with their specialist in the 5 years prior  -previous ischemic heart disease, cerebrovascular disease, serious psychiatric disorders, -taking more than 3 antihypertensive medications  -physical impairments that could make it difficult to practice exercise  -participants in other treatments for weight loss  -participants who had previously participated in the “Living Better” intervention  -no internet access | |
| Gender (n (%)) | f: IG=32 (45.7); CG=28 (45.2) | m: IG=38 (54.3); CG=34 (54.8) |
| Age (M (SD)) | IG=56.2 (9.5) | CG=57.7 (10.7) |
| BMI (M (SD)) | IG=29.6 (3.7) | CG=29.6 (3.2) |
| Intervention | "Living Better": computerized intervention that is self-administered through the internet + own hypertension specialist | |
| Support | None | |
| Control | "Living Better": computerized intervention that is self-administered through the internet + unknown physican | |
| Endpoints and Operationalization | Primary: (1) BMI  Secondary: (1) systolic and diastolic blood pressure, (2) number of antihypertensive drugs, (3) physical activity level (IPAQ-SF), **(4) quality of life** (SF-12 Health Questionnaire) | |
| Study design | RCT | |
| Randomization | 1:1 | |
| Stratification | Age, sex, number of specialist visits | |
| Blinding | Outcome evaluators and data analysts | |
| Observation times | t0=baseline, t1=12 weeks | |
| Sample size | 132 (IG=70; CG=62) | |
| Drop-Out | t1: IG=30; CG=36 | |
| Place of study | Spain | |
| Time of study | 01/2021-06/2021 | |

BMI=body-mass-index | CG=control group | f=female | IG=intervention group | IPAQ=International Physical Activity Questionnaire | m=male | M=mean | n=quantity | RCT=randomized controlled trial | SD=standard deviation

Study characteristics of Gemesi et al. (2023)

| Category | Description | |
| --- | --- | --- |
| Title | Efficacy of an app-based multimodal lifestyle intervention on body weight in persons with obesity: results from a randomized controlled trial | |
| Inclusion Criteria | -adults (women, men)  -age 18-70 years  -BMI 30.0-40.0 kg/m^2^  -no severe diseases (e.g. diagnosed diabetes mellitus, cardiovascular disease, and cancer)  -ownership of a smartphone | |
| Exclusion Criteria | None | |
| Gender (n (%)) | f: IG=57 (67.9); CG=51 (60.7) | m: IG=27 (32.1); CG=33 (39.3) |
| Age (M (SD)) | IG=47.4 (11.5) | CG=46.3 (10.6) |
| BMI (M (SD)) | IG=34.3 (2.5) | CG=34.1 (3.0) |
| Intervention | Application "Oviva Direkt für Adipositas":  -multimodal weight loss intervention program  -three main elements: self-management, self-monitoring, and education  -daily/weekly goals  -entering various data on e.g. nutrition, physical activity, and body weight  -automated feedback in form of weight trajectory curves, reminders (e.g. to enter the current weight at least once a week), motivating notifications, and interpretations  -education content provided on a weekly basis by text, audio, or video format | |
| Support | "Participants were guided through the app installation by a member of the study team. In the first week of app use, participants received a phone call by a qualified coach employed by the app provider. This call aimed to ensure patients’ safety and the appropriate use of the medical device (p.2). | |
| Control | Waiting CG | |
| Endpoints and Operationalization | Primary: (1) weight change (%)  Secondary: (1) **quality of life** (EuroQol (EQ-5D-5L) questionnaire), (2) current, subjective perception of health (EQ VAS score) | |
| Study design | RCT | |
| Randomization | 1:1 | |
| Stratification | None | |
| Blinding | None | |
| Observation times | t0=baseline, t1=12 weeks, t2=24 weeks | |
| Sample size | 181 (IG=91, CG=90) | |
| Drop-Out | t1: IG=28; CG=14 | |
| Place of study | Germany | |
| Time of study | 03/2022-08/2022 | |

BMI=body-mass-index | CG=control group | f=female | IG=intervention group | m=male | M=mean | n=quantity | RCT=randomized controlled trial | SD=standard deviation
