# Supplementary material for: Effectiveness of digital health applications on the quality of life in patients with overweight or obesity: a systematic review
Source: Arch Public Health. 2025 Jan 9;83:3. doi: 10.1186/s13690-024-01474-3 (PMC11715991; doi:10.1186/s13690-024-01474-3)
Supplement: Supplementary file 4 — Additional file 4. Quality assessment of the included studies. [file 13690_2024_1474_MOESM4_ESM.docx]

Additional file 4: Quality assessment of the included studies

Quality assessment of Mensorio et al. (2019)

| Domain | Question | Decision | Justification |
| --- | --- | --- | --- |
| Randomization process | 1.1 Was the allocation sequence random? | Y | "Randomizations were performed using random allocation software (with the program Epidat 4.1)" (p.14) |
|  | 1.2 Was the allocation sequence concealed until participants were enrolled and assigned to interventions? | NI |  |
|  | 1.3 Did baseline differences between intervention groups suggest a problem with the randomization process? | PY | There was a significant difference in baseline BMI (IG=30.08 (2.65); CG=30.15 (2.96); p=0.02) |
| Deviations from intended interventions | 2.1 Were participants aware of their assigned intervention during the trial? | Y | "At 3 months, these participants [CG] were offered the possibility of performing the intervention online." (p.14) |
|  | 2.2 Were carers and people delivering the interventions aware of participants' assigned intervention during the trial? | NI |  |
|  | 2.3 If Y/PY/NI to 2.1 or 2.2: Were there deviations from the intended intervention that arose because of the trial context? | NI |  |
|  | 2.6 Was an appropriate analysis used to estimate the effect of assignment to intervention? | PY | "Bonferroni post-hoc tests were applied, and the intention to-treat (ITT) analysis procedure was used." (p. 16)  ITT was mentioned, but the method of imputation was not explained. |
| Missing outcome data | 3.1 Were data for this outcome available for all, or nearly all, participants randomized? | PN | Drop-Out at primary endpoint regarding QoL: n=15 (14%); IG=12, CG=3 |
|  | 3.2 If N/PN/NI to 3.1: Is there evidence that the result was not biased by missing outcome data? | PN | ITT was mentioned, but the method of imputation was not explained. |
|  | 3.3 If N/PN to 3.2: Could missingness in the outcome depend on its true value? | Y | In IG the drop-out was 12 and in CG only 3. |
|  | 3.4 If Y/PY/NI to 3.3: Is it likely that missingness in the outcome depended on its true value? | PY |  |
| Measurement of the outcome | 4.1 Was the method of measuring the outcome inappropriate? | N | QoL was measured via validated questionnaire (Quality of life (QLI); Spanish version) (p.15) |
|  | 4.2 Could measurement or ascertainment of the outcome have differed between intervention groups? | N | QoL was measured via validated questionnaire in both groups.  "Anthropometric measures were carried out face-to face at the hospital at baseline, 3 months, and 12 months (equivalent dates for both groups). Psychological assessments were carried out online via the Survey Monkey platform. The surveys were e-mailed out to be answered by the patients at baseline, 3 months, 6 months, and 12 months (equivalent dates for both groups)." (p.15) |
|  | 4.3 If N/PN/NI to 4.1 and 4.2: Were outcome assessors aware of the intervention received by study participants? | Y | For participant-reported outcomes, the outcome assessor is the study participant; these were not blinded. |
|  | 4.4 If Y/PY/NI to 4.3: Could assessment of the outcome have been influenced by knowledge of intervention received? | PN | The trial was unblinded. For some participants, the fact that they knew about being in the IG may have influenced their outcome. |
| Selection of the reported result | 5.1 Were the data that produced this result analyzed in accordance with a pre-specified analysis plan that was finalized before unblinded outcome data were available for analysis? | Y | R.M. Baños, M.S. Mensorio, A. Cebolla, E. Rodilla, G. Palomar, J.F. Lisón, C. Botella,  An internet-based self-administered intervention for promoting healthy habits and weight loss in hypertensive people who are overweight or obese: a randomized controlled trial, BMC Cardiovasc. Disord. 15 (2015) |
|  | Is the numerical result being assessed likely to have been selected, on the basis of the results, from...  5.2 ... multiple eligible outcome measurements (e.g. scales, definitions, time points) within the outcome domain? | N | There is no evidence of this. |
|  | Is the numerical result being assessed likely to have been selected, on the basis of the results, from...  5.3 ... multiple eligible analyses of the data? | N | There is no evidence of this. |

N=no | NI=no information | PN=partly no | PY=partly yes | Y=yes

Quality assessment of Mangieri et al. (2019)

| Domain | Question | Decision | Justification |
| --- | --- | --- | --- |
| Randomization process | 1.1 Was the allocation sequence random? | NI |  |
|  | 1.2 Was the allocation sequence concealed until participants were enrolled and assigned to interventions? | NI |  |
|  | 1.3 Did baseline differences between intervention groups suggest a problem with the randomization process? | N | There were no baseline differences between IG and CG. (p.3) |
| Deviations from intended interventions | 2.1 Were participants aware of their assigned intervention during the trial? | Y | "This single institution prospective randomized control trial compared the use of a mHealth application versus standard weight loss monitoring after bariatric surgery." (p.2) |
|  | 2.2 Were carers and people delivering the interventions aware of participants' assigned intervention during the trial? | Y | "The research coordinator had access to each MyFitnessPal© application profile […]" (p.2) |
|  | 2.3 If Y/PY/NI to 2.1 or 2.2: Were there deviations from the intended intervention that arose because of the trial context? | PY | "The research coordinator had access to each MyFitnessPal© application profile and would contact patients who did not record any data in the application for greater than 48 h." (p.2) |
|  | 2.4 If Y/PY to 2.3: Were these deviations likely to have affected the outcome? | PY | These deviations likely to have affected QoL. |
|  | 2.5. If Y/PY/NI to 2.4: Were these deviations from intended intervention balanced between groups? | N | These deviations from intended intervention were only relevant for IG. |
|  | 2.6 Was an appropriate analysis used to estimate the effect of assignment to intervention? | NI |  |
|  | 2.7 If N/PN/NI to 2.6: Was there potential for a substantial impact (on the result) of the failure to analyze participants in the group to which they were randomized? | N | There was no potential for a substantial impact on QoL of the failure to analyze participants in the group to which they were randomized. |
| Missing outcome data | 3.1 Were data for this outcome available for all, or nearly all, participants randomized? | Y | Drop-Out=0 |
| Measurement of the outcome | 4.1 Was the method of measuring the outcome inappropriate? | N | QoL was measured via validated questionnaire (RAND-36). (p.2) |
|  | 4.2 Could measurement or ascertainment of the outcome have differed between intervention groups? | NI |  |
|  | 4.3 If N/PN/NI to 4.1 and 4.2: Were outcome assessors aware of the intervention received by study participants? | Y | For participant-reported outcomes, the outcome assessor is the study participant; these were not blinded. |
|  | 4.4 If Y/PY/NI to 4.3: Could assessment of the outcome have been influenced by knowledge of intervention received? | PN | The trial was unblinded. For some participants, the fact that they knew about being in the IG may have influenced their outcome. |
| Selection of the reported result | 5.1 Were the data that produced this result analyzed in accordance with a pre-specified analysis plan that was finalized before unblinded outcome data were available for analysis? | PY | The publication contains a short statistical analysis plan (p.2-3). However, no additional study protocol was cited. |
|  | Is the numerical result being assessed likely to have been selected, on the basis of the results, from...  5.2 ... multiple eligible outcome measurements (e.g. scales, definitions, time points) within the outcome domain? | NI |  |
|  | Is the numerical result being assessed likely to have been selected, on the basis of the results, from...  5.3 ... multiple eligible analyses of the data? | NI |  |

N=no | NI=no information | PN=partly no | PY=partly yes | Y=yes

Quality assessment of Kraschnweski et al. (2011)

| Domain | Question | Decision | Justification |
| --- | --- | --- | --- |
| Randomization process | 1.1 Was the allocation sequence random? | Y | "Randomization took place at the baseline visit using concealed envelopes." (p. 611) |
|  | 1.2 Was the allocation sequence concealed until participants were enrolled and assigned to interventions? | NI |  |
|  | 1.3 Did baseline differences between intervention groups suggest a problem with the randomization process? | PN | "Baseline characteristics of participants are shown in Table 1; no signifıcant group differences were observed." (p.612)  Difference in gender and self-reported health status (p.612) |
| Deviations from intended interventions | 2.1 Were participants aware of their assigned intervention during the trial? | Y | "If randomized to the AchieveTogether intervention, participants were e-mailed a link to the website. If randomized to wait-list control condition, participants were notified that they would receive website access after 12 weeks." (p.611) |
|  | 2.2 Were carers and people delivering the interventions aware of participants' assigned intervention during the trial? | Y | "All participants were shown in person how to use the AchieveTogether weight-loss website at the baseline visit." (p.611) |
|  | 2.3 If Y/PY/NI to 2.1 or 2.2: Were there deviations from the intended intervention that arose because of the trial context? | NI |  |
|  | 2.6 Was an appropriate analysis used to estimate the effect of assignment to intervention? | N | No ITT was used, and dropouts were not analyzed. |
|  | 2.7 If N/PN/NI to 2.6: Was there potential for a substantial impact (on the result) of the failure to analyze participants in the group to which they were randomized? | Y | There was a drop-out of 14% in IG and 10% in CG. It is possible that individuals dropped out due to the intervention design (or other factors). |
| Missing outcome data | 3.1 Were data for this outcome available for all, or nearly all, participants randomized? | PN | Drop-Out at primary endpoint regarding QoL: n=12 (12%); IG=7, CG=5 |
|  | 3.2 If N/PN/NI to 3.1: Is there evidence that the result was not biased by missing outcome data? | N | No ITT was used, and dropouts were not analyzed. |
|  | 3.3 If N/PN to 3.2: Could missingness in the outcome depend on its true value? | Y | In IG the drop-out was 7 and in CG only 5. |
|  | 3.4 If Y/PY/NI to 3.3: Is it likely that missingness in the outcome depended on its true value? | PY |  |
| Measurement of the outcome | 4.1 Was the method of measuring the outcome inappropriate? | N | "Previously validated questionnaires were administered including the Block 2005 Food Frequency Questionnaire17,18 to assess caloric intake and the Impact of Weight on Quality of Life questionnaire." (p.612) |
|  | 4.2 Could measurement or ascertainment of the outcome have differed between intervention groups? | NI |  |
|  | 4.3 If N/PN/NI to 4.1 and 4.2: Were outcome assessors aware of the intervention received by study participants? | Y | For participant-reported outcomes, the outcome assessor is the study participant; these were not blinded. |
|  | 4.4 If Y/PY/NI to 4.3: Could assessment of the outcome have been influenced by knowledge of intervention received? | PN | The trial was unblinded. For some participants, the fact that they knew about being in the IG may have influenced their outcome. |
| Selection of the reported result | 5.1 Were the data that produced this result analyzed in accordance with a pre-specified analysis plan that was finalized before unblinded outcome data were available for analysis? | PY | The publication contains a short statistical analysis plan (p.612). However, no additional study protocol was cited. |
|  | Is the numerical result being assessed likely to have been selected, on the basis of the results, from...  5.2 ... multiple eligible outcome measurements (e.g. scales, definitions, time points) within the outcome domain? | N | There is no evidence of this. |
|  | Is the numerical result being assessed likely to have been selected, on the basis of the results, from...  5.3 ... multiple eligible analyses of the data? | N | There is no evidence of this. |

N=no | NI=no information | PN=partly no | PY=partly yes | Y=yes

Quality assessment of Mc Connon et al. (2007)

| Domain | Question | Decision | Justification |
| --- | --- | --- | --- |
| Randomization process | 1.1 Was the allocation sequence random? | Y | "A computer-generated randomization procedure was employed, using the software package 'minim'". (p.3) |
|  | 1.2 Was the allocation sequence concealed until participants were enrolled and assigned to interventions? | NI |  |
|  | 1.3 Did baseline differences between intervention groups suggest a problem with the randomization process? | N | "Preliminary analysis showed no significant differences between the two groups at baseline". (p.4) |
| Deviations from intended interventions | 2.1 Were participants aware of their assigned intervention during the trial? | Y | "Due to the pragmatic nature of the trial and the intervention being evaluated, it was not possible to blind either the participants or researchers to the group assignment" (p.3) |
|  | 2.2 Were carers and people delivering the interventions aware of participants' assigned intervention during the trial? | Y |  |
|  | 2.3 If Y/PY/NI to 2.1 or 2.2: Were there deviations from the intended intervention that arose because of the trial context? | N | "The website aimed to offer a low maintenance weight loss tool with limited professional support." (p.7) |
|  | 2.6 Was an appropriate analysis used to estimate the effect of assignment to intervention? | N | Results of ANCOVA with data imputation and responder analysis were presented for BMI but not for quality of life. |
|  | 2.7 If N/PN/NI to 2.6: Was there potential for a substantial impact (on the result) of the failure to analyze participants in the group to which they were randomized? | Y | There was a drop-out of 48,6% in IG and 70% in CG. It is possible that individuals dropped out due to the intervention design (or other factors). |
| Missing outcome data | 3.1 Were data for this outcome available for all, or nearly all, participants randomized? | N | "Measurements were obtained for 69% (n=152) of the sample at 6 months and for 59% (n=131) at 12 months, equating to an attrition rate of 31% at six months and 41% at 12 months." (p.4) |
|  | 3.2 If N/PN/NI to 3.1: Is there evidence that the result was not biased by missing outcome data? | N | There is no evidence of this. |
|  | 3.3 If N/PN to 3.2: Could missingness in the outcome depend on its true value? | Y | In IG the drop-out was 54 and in CG 77. |
|  | 3.4 If Y/PY/NI to 3.3: Is it likely that missingness in the outcome depended on its true value? | PY |  |
| Measurement of the outcome | 4.1 Was the method of measuring the outcome inappropriate? | N | "Quality of life was assessed using the EuroQol questionnaire, a short, self-administered questionnaire which was also used in the cost effectiveness analysis." (p.3) |
|  | 4.2 Could measurement or ascertainment of the outcome have differed between intervention groups? | NI |  |
|  | 4.3 If N/PN/NI to 4.1 and 4.2: Were outcome assessors aware of the intervention received by study participants? | Y | For participant-reported outcomes, the outcome assessor is the study participant; these were not blinded. |
|  | 4.4 If Y/PY/NI to 4.3: Could assessment of the outcome have been influenced by knowledge of intervention received? | PN | The trial was unblinded. For some participants, the fact that they knew about being in the IG may have influenced their outcome. |
| Selection of the reported result | 5.1 Were the data that produced this result analyzed in accordance with a pre-specified analysis plan that was finalized before unblinded outcome data were available for analysis? | PY | The publication contains a short statistical analysis plan (p.3). However, no additional study protocol was cited. |
|  | Is the numerical result being assessed likely to have been selected, on the basis of the results, from...  5.2 ... multiple eligible outcome measurements (e.g. scales, definitions, time points) within the outcome domain? | NI |  |
|  | Is the numerical result being assessed likely to have been selected, on the basis of the results, from...  5.3 ... multiple eligible analyses of the data? | NI |  |

N=no | NI=no information | PN=partly no | PY=partly yes | Y=yes

Quality assessment of Roth et al. (2023)

| Domain | Question | Decision | Justification |
| --- | --- | --- | --- |
| Randomization process | 1.1 Was the allocation sequence random? | PY | "Eligible participants were randomly assigned by a trained study coordinator to the IG or CG using a sequential, stratified randomization approach to ensure equal distribution of potentially moderating variables." (p.1301-1302) |
|  | 1.2 Was the allocation sequence concealed until participants were enrolled and assigned to interventions? | NI |  |
|  | 1.3 Did baseline differences between intervention groups suggest a problem with the randomization process? | PN | "Overall, both groups were comparable regarding demographic data and initial values of end points." (p.1305)  Difference in education level, shift work and marital status (p.1305) |
| Deviations from intended interventions | 2.1 Were participants aware of their assigned intervention during the trial? | Y | "Study participants were not blinded because of the nature of the intervention." (p.1302) |
|  | 2.2 Were carers and people delivering the interventions aware of participants' assigned intervention during the trial? | Y | "Similarly, study personnel were unblinded because they provided all relevant information to IG participants and, if necessary, assisted with account activation." (p.1302) |
|  | 2.3 If Y/PY/NI to 2.1 or 2.2: Were there deviations from the intended intervention that arose because of the trial context? | PY | "Structured information on whether participants of the IG used additional programs is lacking and will be assessed in future studies." (p.1308) |
|  | 2.4 If Y/PY to 2.3: Were these deviations likely to have affected the outcome? | PY | These deviations likely to have affected QoL. |
|  | 2.5. If Y/PY/NI to 2.4: Were these deviations from intended intervention balanced between groups? | NI |  |
|  | 2.6 Was an appropriate analysis used to estimate the effect of assignment to intervention? | Y | "Along with descriptive statistics of baseline data, intention-to-treat (ITT) analyses as well as per-protocol (PP) analyses were conducted for all end points." (p.1303) |
| Missing outcome data | 3.1 Were data for this outcome available for all, or nearly all, participants randomized? | PN | Drop-Out at primary endpoint regarding QoL: n=15 (10.7%); IG=9, CG=6 |
|  | 3.2 If N/PN/NI to 3.1: Is there evidence that the result was not biased by missing outcome data? | Y | ITT was used |
| Measurement of the outcome | 4.1 Was the method of measuring the outcome inappropriate? | N | "To measure general, disease-independent QoL, we used the WHOQOL-BREF questionnaire [...]" (p.1303) |
|  | 4.2 Could measurement or ascertainment of the outcome have differed between intervention groups? | N | "Demographic data and psychological measures (well-being and QoL) were assessed via online surveys administered using SoSci." (p.1302) |
|  | 4.3 If N/PN/NI to 4.1 and 4.2: Were outcome assessors aware of the intervention received by study participants? | Y | For participant-reported outcomes, the outcome assessor is the study participant; these were not blinded. |
|  | 4.4 If Y/PY/NI to 4.3: Could assessment of the outcome have been influenced by knowledge of intervention received? | PN | The trial was unblinded. For some participants, the fact that they knew about being in the IG may have influenced their outcome. |
| Selection of the reported result | 5.1 Were the data that produced this result analyzed in accordance with a pre-specified analysis plan that was finalized before unblinded outcome data were available for analysis? | Y | The trial was conducted in accordance with the Declaration of Helsinki and was registered at the German Clinical Trials Register (DRKS00024415)." (p.1301) |
|  | Is the numerical result being assessed likely to have been selected, on the basis of the results, from...  5.2 ... multiple eligible outcome measurements (e.g. scales, definitions, time points) within the outcome domain? | N | There is no evidence of this. |
|  | Is the numerical result being assessed likely to have been selected, on the basis of the results, from...  5.3 ... multiple eligible analyses of the data? | N | There is no evidence of this. |

N=no | NI=no information | PN=partly no | PY=partly yes | Y=yes

Quality assessment of Múzquiz-Barberá et al. (2023)

| Domain | Question | Decision | Justification |
| --- | --- | --- | --- |
| Randomization process | 1.1 Was the allocation sequence random? | Y | "Before the start of the trial, an independent researcher unaware of the study characteristics generated a random sequence using a computerized random number generator; this was concealed from all the other study investigators throughout the entire study period." (p.3) |
|  | 1.2 Was the allocation sequence concealed until participants were enrolled and assigned to interventions? | Y | "Upon enrollment in the study and after completing the primary and secondary outcome measures, the participants (N=132) were randomly assigned either to the control (n=62) or the experimental group (n=70)." (p.3) |
|  | 1.3 Did baseline differences between intervention groups suggest a problem with the randomization process? | PN | Difference in weight, physical activity level and quality of life (p.5) |
| Deviations from intended interventions | 2.1 Were participants aware of their assigned intervention during the trial? | Y | "It was impossible to mask the group allocation to the participants [...]" (p.3) |
|  | 2.2 Were carers and people delivering the interventions aware of participants' assigned intervention during the trial? | NI |  |
|  | 2.3 If Y/PY/NI to 2.1 or 2.2: Were there deviations from the intended intervention that arose because of the trial context? | PY | "Secondly, this single-center clinical trial only involved one doctor per arm, and therefore is potentially confounded by their personal characteristics that could have influenced the outcomes." (p.7) |
|  | 2.4 If Y/PY to 2.3: Were these deviations likely to have affected the outcome? | PY |  |
|  | 2.5. If Y/PY/NI to 2.4: Were these deviations from intended intervention balanced between groups? | NI |  |
|  | 2.6 Was an appropriate analysis used to estimate the effect of assignment to intervention? | PN | "Despite these two differences (lower adherence and increased losses after the intervention), the results of the intention-to-treat statistical analysis showed that all participants experienced benefits." (p.6) This is remarkable given the no-effect hypothesis for those that dropped out. |
|  | 2.7 If N/PN/NI to 2.6: Was there potential for a substantial impact (on the result) of the failure to analyse participants in the group to which they were randomized? | PY | There was a drop-out of 43% in IG and 58% in CG. It is possible that individuals dropped out due to the intervention design (or other factors). |
| Missing outcome data | 3.1 Were data for this outcome available for all, or nearly all, participants randomized? | N | Drop-Out at primary endpoint regarding QoL: n=66 (50%); IG=30, CG=36 |
|  | 3.2 If N/PN/NI to 3.1: Is there evidence that the result was not biased by missing outcome data? | PY | ITT was conducted but data imputation was not described. |
| Measurement of the outcome | 4.1 Was the method of measuring the outcome inappropriate? | N | "The SF-12 Health Questionnaire (a reduced version of the SF-36) was applied to measure quality of life." (p.3) |
|  | 4.2 Could measurement or ascertainment of the outcome have differed between intervention groups? | N | "Furthermore, the variables listed below were recorded before and after the intervention, through the same platform as the intervention program." (p.3) |
|  | 4.3 If N/PN/NI to 4.1 and 4.2: Were outcome assessors aware of the intervention received by study participants? | Y | For participant-reported outcomes, the outcome assessor is the study participant; these were not blinded. |
|  | 4.4 If Y/PY/NI to 4.3: Could assessment of the outcome have been influenced by knowledge of intervention received? | PN | The trial was unblinded. For some participants, the fact that they knew about being in the IG may have influenced their outcome. |
| Selection of the reported result | 5.1 Were the data that produced this result analyzed in accordance with a pre-specified analysis plan that was finalized before unblinded outcome data were available for analysis? | Y | "This was a prospective, single-center, clinical trial (registered at ClinicalTrials.gov: NCT04426877) with balanced randomization (1:1)." (p.2) |
|  | Is the numerical result being assessed likely to have been selected, on the basis of the results, from...  5.2 ... multiple eligible outcome measurements (e.g. scales, definitions, time points) within the outcome domain? | N | There is no evidence of this. |
|  | Is the numerical result being assessed likely to have been selected, on the basis of the results, from...  5.3 ... multiple eligible analyses of the data? | N | There is no evidence of this. |

N=no | NI=no information | PN=partly no | PY=partly yes | Y=yes

Quality assessment of Gemesi et al. (2023)

| Domain | Question | Decision | Justification |
| --- | --- | --- | --- |
| Randomization process | 1.1 Was the allocation sequence random? | Y | "Included participants were randomized to two study groups (ADHOC and EXPECT) with an allocation ratio of 1:1 using the Stat Trek Random Number Generator." (p.2) |
|  | 1.2 Was the allocation sequence concealed until participants were enrolled and assigned to interventions? | NI |  |
|  | 1.3 Did baseline differences between intervention groups suggest a problem with the randomization process? | N | "Participants of the two groups did not differ significantly in any of the baseline parameters (all p > 0.05)." (p.3) |
| Deviations from intended interventions | 2.1 Were participants aware of their assigned intervention during the trial? | Y | "In the beginning of the weight loss intervention (for ADHOC at V1, for EXPECT at V2 after 12 weeks of “waiting” period) participants were guided through the app installation by a member of the study team." p.2 |
|  | 2.2 Were carers and people delivering the interventions aware of participants' assigned intervention during the trial? | Y |  |
|  | 2.3 If Y/PY/NI to 2.1 or 2.2: Were there deviations from the intended intervention that arose because of the trial context? | NI |  |
|  | 2.6 Was an appropriate analysis used to estimate the effect of assignment to intervention? | N | For QoL, only completer-analysis is presented. |
|  | 2.7 If N/PN/NI to 2.6: Was there potential for a substantial impact (on the result) of the failure to analyse participants in the group to which they were randomized? | Y | There was a drop-out of 30.8% in IG and 15.6% in CG. It is possible that individuals dropped out due to the intervention design (or other factors). |
| Missing outcome data | 3.1 Were data for this outcome available for all, or nearly all, participants randomized? | N | Drop-Out at primary endpoint regarding QoL: n=42 (23.2%); IG=28, CG=14 |
|  | 3.2 If N/PN/NI to 3.1: Is there evidence that the result was not biased by missing outcome data? | N | ITT was not used |
|  | 3.3 If N/PN to 3.2: Could missingness in the outcome depend on its true value? | Y | In IG the drop-out was 28 and in CG 14. |
|  | 3.4 If Y/PY/NI to 3.3: Is it likely that missingness in the outcome depended on its true value? | PY |  |
| Measurement of the outcome | 4.1 Was the method of measuring the outcome inappropriate? | N | "Health-related quality of life was assessed at all three study visits  through the validated EuroQol (EQ-5D-5L) questionnaire [...]." (p.3) |
|  | 4.2 Could measurement or ascertainment of the outcome have differed between intervention groups? | NI |  |
|  | 4.3 If N/PN/NI to 4.1 and 4.2: Were outcome assessors aware of the intervention received by study participants? | NI | For participant-reported outcomes, the outcome assessor is the study participant; these were not blinded. |
|  | 4.4 If Y/PY/NI to 4.3: Could assessment of the outcome have been influenced by knowledge of intervention received? | PN | The trial was unblinded. For some participants, the fact that they knew about being in the IG may have influenced their outcome. |
| Selection of the reported result | 5.1 Were the data that produced this result analyzed in accordance with a pre-specified analysis plan that was finalized before unblinded outcome data were available for analysis? | Y | "The study protocol has been approved by the local ethics committee (vote number: 45/22 S-NP) and was registered at the German Clinical Trials Register (Registration number: DRKS00025291)." (p.2) |
|  | Is the numerical result being assessed likely to have been selected, on the basis of the results, from...  5.2 ... multiple eligible outcome measurements (e.g. scales, definitions, time points) within the outcome domain? | N | There is no evidence of this. |
|  | Is the numerical result being assessed likely to have been selected, on the basis of the results, from...  5.3 ... multiple eligible analyses of the data? | N | There is no evidence of this. |

N=no | NI=no information | PN=partly no | PY=partly yes | Y=yes
