# Supplementary material for: Effectiveness of digital health applications on the quality of life in patients with overweight or obesity: a systematic review
Source: Arch Public Health. 2025 Jan 9;83:3. doi: 10.1186/s13690-024-01474-3 (PMC11715991; doi:10.1186/s13690-024-01474-3)
Supplement: Supplementary file 5 — Additional file 5. Study results on BMI and weight. [file 13690_2024_1474_MOESM5_ESM.docx]

Additional file 5: Study results on BMI and weight

Study results of the included studies – BMI and weight

| Author (Year) | Statistical measure | Primary endpoint | Observation time | Results IG | Results CG | p-value | Risk of Bias |
| --- | --- | --- | --- | --- | --- | --- | --- |
| Mensorio et al. (2019)  *CG received the digital intervention after 3 months*  *ANOVA, ITT* | M (SD) | BMI | t0 (baseline) | 30.08 (2.65) | 30.15 (2.96) | .02 | High |
|  |  |  | t1 (3 months)^A^ | 29.64 (2.62) | 30.48 (3.04) | <.01 |  |
|  |  |  | t3 (12 months) | 29.8 (2.46) | 29.8 (3.5) | n.r. |  |
| Mangieri et al. (2019)  *ANOVA* | M (SD) | %EWL | t0 (baseline) | 40.07 (6.9) | 37.67 (6.9) | .37 | High |
|  |  |  | t1 (12 months) | 81.41 (6.9) | 74.4 (8.0) | .0468 |  |
|  |  |  | t2 (24 months)^A^ | 71.4 (6.8) | 59.10 (9.9) | .0078 |  |
|  |  | %EBL | t0 (baseline) | 13.58 (2.22) | 13.50 (2.27) | .93 |  |
|  |  |  | t1 (12 months) | 32.15 (2.26) | 28.02 (2.26) | <.001 |  |
|  |  |  | t2 (24 months)^A^ | 27.87 (2.20) | 25.39 (2.20) | .0479 |  |
| Kraschnewski et al. (2011)  *ANCOVA* | M [95% CI] | Δ Weight in kg | t0 (baseline) | 92.2 (13.6) | 94.2 (15.2) | .48 | High |
|  |  |  | t1 (3 months)^A^ | -1.4 [-2.2, -0.5] | 0.6 [-0.3, 1.4] | <.01 |  |
| Mc Connon et al. (2007)  *ANCOVA* | M (SD) | Δ Weight in kg | t0 (baseline) | 98.9 (17.7) | 97.9 (17.1) | n.r. | High |
|  |  |  | t1 (12 months)^A^ | MD=0.6 [-1.4 to 2.5] | | .56 |  |
| Roth et al. (2023)  *ITT* | M (SD) | Δ Weight in (kg) in % | t0 (baseline) | 102.2 (12.3) | 103.0 (14.6) | n.r. | High |
|  |  |  | t1 (12 months)^A^ | -7.75 [-9.66 to -5.84] | 0.00 [-1.98 to 1.99] | <.001 |  |
|  |  |  |  | -7.75 [-9.61 to -5.85] | |  |  |
| Múzquiz-Barberá et al (2023)  *ANOVA, ITT* | M (SD) | BMI | t0 (baseline) | 29.6 (3.7) | 29.6 (3.2) | .972 | High |
|  |  |  | t1 (3 months)^A^ | 29.2 (3.7) | 29.3 (3.5) | .852 |  |
| Gemesi et al. (2023)  *Completers-Analysis* | M (SD) | Δ Weight in (kg) in % | t0 (baseline) | 100.8 (12.1) | 100.6 (4.8) | .93 | High |
|  |  |  | t1 (3 months)^A^ | −3.2 (3.0) | −0.3 (2.6) | <.001 |  |
|  |  |  |  | MD=−2.9 [−3.8; −1.9] | |  |  |

^A^ primary survey time point

ANCOVA=analysis of covariance | ANOVA=analysis of variance | BMI=body-mass-index | CG=control group | CI=confidence interval | EBL=excess BMI loss (=preoperative BMI-current BMI)/(preoperative BMI-25)*100 | EWL=excess bodyweight loss (=preoperative weight-current weight)/(preoperative weight-ideal weight))*100) | IG=intervention group | IQR=interquartile range | ITT=intention-to-treat | M=mean | MD=mean difference | n.a.=not applicable | n.r.=not reported | SD=standard deviation | QoL=quality of life
